# Cdc42/Rac Interactive Binding Containing Effector Proteins in Unicellular Protozoans With Reference to Human Host: Locks of the Rho Signaling

**DOI:** 10.3389/fgene.2022.781885

**Published:** 2022-02-02

**Authors:** Preeti Umarao, Pragyan Parimita Rath, Samudrala Gourinath

**Affiliations:** Structural Biology Lab, School of Life Sciences, Jawaharlal Nehru University, New Delhi, India

**Keywords:** amoebozoa, CRIB, PAK, CDC42/RAC, WASP, coronin

## Abstract

Small GTPases are the key to actin cytoskeleton signaling, which opens the lock of effector proteins to forward the signal downstream in several cellular pathways. Actin cytoskeleton assembly is associated with cell polarity, adhesion, movement and other functions in eukaryotic cells. Rho proteins, specifically Cdc42 and Rac, are the primary regulators of actin cytoskeleton dynamics in higher and lower eukaryotes. Effector proteins, present in an inactive state gets activated after binding to the GTP bound Cdc42/Rac to relay a signal downstream. Cdc42/Rac interactive binding (CRIB) motif is an essential conserved sequence found in effector proteins to interact with Cdc42 or Rac. A diverse range of Cdc42/Rac and their effector proteins have evolved from lower to higher eukaryotes. The present study has identified and further classified CRIB containing effector proteins in lower eukaryotes, focusing on parasitic protozoans causing neglected tropical diseases and taking human proteins as a reference point to the highest evolved organism in the evolutionary trait. Lower eukaryotes’ CRIB containing proteins fall into conventional effector molecules, PAKs (p21 activated kinase), Wiskoit-Aldrich Syndrome proteins family, and some have unique domain combinations unlike any known proteins. We also highlight the correlation between the effector protein isoforms and their selective specificity for Cdc42 or Rac proteins during evolution. Here, we report CRIB containing effector proteins; ten in *Dictyostelium* and *Entamoeba*, fourteen in *Acanthamoeba*, one in *Trypanosoma* and *Giardia*. CRIB containing effector proteins that have been studied so far in humans are potential candidates for drug targets in cancer, neurological disorders, and others. Conventional CRIB containing proteins from protozoan parasites remain largely elusive and our data provides their identification and classification for further in-depth functional validations. The tropical diseases caused by protozoan parasites lack combinatorial drug targets as effective paradigms. Targeting signaling mechanisms operative in these pathogens can provide greater molecules in combatting their infections.

## Introduction

Cellular functions are a cumulative outcome of various signaling pathways that involve a downstream activation of a series of protein molecules. Cytoskeletal organization and dynamics critically regulate cell movement and migration, proliferation, adhesion, differentiation, and vesicular trafficking (Sackmann, 2015). Molecular switches are crucial proteins, which interact with their effectors to activate a signaling cascade. A varsity of small GTPase molecules is present in the cell where Rho family proteins are regulatory molecules for actin cytoskeleton dynamics (Hall, 1998).

Cdc42, Rho, and Rac are Rho family proteins’ subfamilies, a Ras superfamily subgroup (Wennerberg et al., 2005). These proteins oscillate between an active GTP-bound and inactive GDP-bound states as molecular switches in the cell (Vetter and Wittinghofer, 2001). In humans, several studies have characterized Cdc42 (filopodia formation), Rac1 (lamellipodia formation), and RhoA (establishment of stress fiber) members of the Rho family (Jordan and Canman, 2012; Ridley, 2015; Narumiya and Thumkeo, 2018). Cdc42 (Cell division control protein 42) was the first member to be reported (Shinjo et al., 1990; Marks and Kwiatkowski, 1996) with its profound functions in; cell polarity (in yeast), cell morphology regulation, motility, mammalian cell-cycle progression and induction of malignant cell transformation (summary by (Wu et al., 2000)). Rac1 modulates cytoskeleton in multiple cellular functions like phagocytosis, neural polarization and axonal growth, mesenchymal-like migration, and cellular growth and differentiation (summary by (Reijnders et al., 2017)). The signaling cascade of Cdc42 and Rac proteins recognize a consensus motif in downstream proteins for specific binding. Thus, these proteins were coined as CRIB (Cdc42/Rac interactive binding) effector proteins of the Rho family. The conserved motif is a 16 amino acid sequence “I-S-X-P-(X)_2-4_-F-X-H-X-X-H-V-G”, with eight core amino acids first identified by Burbelo in 1995 (Burbelo et al., 1995). Interestingly, effector proteins with one or two variations within the core sequence can still bind to Cdc42/Rac (Owen et al., 2000). Biophysical studies elucidated that the CRIB motif is essential for interaction with GTP-Cdc42/Rac, but not adequate for high-affinity binding (Rudolph et al., 1998; Thompson et al., 1998). The binding region of CRIB effector protein is thus, also called as gtpase binding domain (GBD) (Rudolph et al., 1998). Subsequently, when the motif, including a more extended sequence region was found in p21-activated kinase (PAK) CRIB effector protein, it was known as p21-binding domain (PBD) (Thompson et al., 1998).

The potential CRIB effector proteins were then categorically separated via different signaling pathways activated by Cdc42/Rac (Neudauer et al., 1998; Bishop and Hall, 2000; Phillips et al., 2008). Cdc42 and Rac activate effector proteins to signal downstream to function on actin, SRF and NF-kB (transcription factor), JNK and p38 (MAP kinase pathway), G1-cell cycle progression, cell-cell contact, and transformation (Bishop and Hall, 2000). NADPH oxidase complex (present only in professional phagocytic cells) and secretin (only in mast cells) signaling pathways get explicitly activated by binding Rac to CRIB effector protein. Similarly, the cell-polarity signaling pathway also involves the CRIB effector proteins, triggered only by Cdc42 (Neudauer et al., 1998; Bishop and Hall, 2000; Vlahou and Rivero, 2006). The effector proteins of Cdc42/Rac are diverse in domain architecture and function, which includes Ser/Thr kinase, cytosolic Tyrosine kinase, actin-associated proteins, adaptor proteins, and miscellaneous (Owen and Mott, 2018). Ser/Thr kinases include PAKs, myotonic dystrophy kinase-related Cdc42 binding kinases (MRCKs) and mixed-lineage kinases (MLKs) family. In Tyrosine kinase, only one family of activated Cdc42-associated Tyrosine kinase (ACK) contain a CRIB motif (Bishop and Hall, 2000; Pirone et al., 2000; Owen and Mott, 2018). Actin associated proteins include Wiskott Aldrich syndrome protein (WASP), WASP-like verproline-homologous protein (WAVE), IQ motif-containing GTPase-activating proteins (IQGAP) and formin families of proteins (Bishop and Hall, 2000; Owen and Mott, 2018). Partitioning defective (PAR) proteins belong to adaptor proteins of cytoskeleton assembly. Small protein effector of Cdc42 (SPEC) and Cdc42 effector protein (CEP)/Binder of Rho GTPases (Borg) (Joberty et al., 1999; Hirsch et al., 2001) family of proteins fall under the miscellaneous group because they have not been designated under any specific classification (Owen and Mott, 2018).

The diverse signaling range and protein families project the necessity of CRIB effector proteins. In humans, CRIB containing proteins have been explored structurally and functionally in depth (Pirone et al., 2001; Cotteret and Chernoff, 2002). CRIB containing effector proteins in humans belong to nine distinct families, four kinase families and five non-kinase families (Bishop and Hall, 2000). Six PAKs (PAK1-6), three MRCKs, two MLKs, three PAR-6, two WASP (WASP and N-WASP) (Kuspa and Loomis, 2006), and two SPEC (Pirone et al., 2000), which are commonly known families of Cdc42/Rac effectors. Additionally, two unique families, Gene33 (one member) (Makkinje et al., 2000) and CEP/Borg (five members) (Joberty et al., 1999; Hirsch et al., 2001) are present only in humans. In plants, eleven Rop-interactive CRIB motif-containing protein (RIC) effectors, Ric1 to Ric11, are present, involved in cell growth like CRIB effector protein in metazoans (Wu et al., 2001; Cotteret and Chernoff, 2002; Fu et al., 2005).

CRIB effector proteins present in complex and multicellular eukaryotes such as worms, flies, and frogs are mammalian homologues (Pirone et al., 2001). Nine proteins in *D. melanogaster*; DmMLK (Teramoto et al., 1996), DmGEK (MRCK) (Luo et al., 1997; Leung et al., 1998), DmACK (Manser et al., 1995; Clemens et al., 2000), DmPAK1-3 are kinases (Mentzel and Raabe, 2005) and DmWASP (Symons et al., 1996; Miki et al., 1998a), DmPAR-6 (Qiu et al., 2000), DmSPEC (Pirone et al., 2000) are non-kinase. All nine proteins are architecturally homologous to humans and perform alike functions. Eight proteins in *C. elegans*; CePAK1 and CePAK2 (Lucanic and Cheng, 2008), are PAK-like kinases, CeACK and CeMRCK are ACK (Manser et al., 1995), and MRCK (Leung et al., 1998) homologs, respectively, and other four belonging to non-kinases are CeWASP (Symons et al., 1996; Miki et al., 1998a; Zhang and Kubiseski, 2010), CePAR-6 (Qiu et al., 2000), F09F7.5 and T23G5.3 (Pirone et al., 2001; Cotteret and Chernoff, 2002). All four non-kinase Cdc42/Rac effectors share no familiar domain characteristic features except for the consensus CRIB/GBD domain. WASP and Par-6 participate in cell polarity through actin organization, while F09F7.5 and T23G5.3 are novel proteins with no homologues in humans or flies (Pirone et al., 2001). In simple eukaryotes such as yeast, there are only five such proteins, Ste20, Skm1, and Cla4, within the PAK family, and Gic1 and Gic2 (Brown et al., 1997), which are non-kinases homologous to plants, and not mammalian CRIB effector proteins. The characteristically different Gics might have a specialized role in cytoskeleton modulation during cell wall assembly.

The extended repertoire of Cdc42/Rac effector proteins in humans indicates a more complex mechanism for extracellular signals to reach Rho GTPases (Cdc42 and Rac) compared to *Drosophila*. Nevertheless, in worms, each group of effectors have a single protein to perform a related signaling function, while in yeast, only the PAK family is present. The trend highlights that complexity of an organism is correlated with the extension of protein members in each effector protein group. Evolutionarily, the presence of CRIB containing effector proteins in plants interestingly points an ancient origin of the CRIB motif. Possibly, the CRIB motif associated with an array of Rho signaling proteins during evolution.

The CRIB containing effector proteins from unicellular eukaryotes are not structurally characterized or classified. Here, we present the identification of effector proteins in unicellular eukaryotes, offering exciting insights into their evolutionary connection to higher eukaryotes’ signaling. Many unicellular eukaryotes organisms are pathogenic and cause dire health challenges with high prevalence. In the new world era, the treatments available for diseases like amoebiasis, sleeping sickness and protozoan parasitic diseases are not very effective and need more attention for targeted drug research (Baker et al., 2013; Diaz et al., 2014; Gonzales et al., 2019; Carrero et al., 2020). The CRIB domain effector proteins, which are barely known and investigated in protozoan parasites can be a prospective candidate for drug research as they are integral to cellular signaling cascades during parasitic pathogenesis. In humans, PAK and WASP family proteins have been thoroughly inspected, both functionally and biochemically, and they proved to be potential targets against cancer, neurodegenerative and cardiovascular diseases (Kichina et al., 2010; Li et al., 2010; Zhao and Manser, 2010; Llorens et al., 2013; Dammann et al., 2018). Rho signaling effectors are the mediators of cytoskeletal dynamics in higher organisms and are crucial for unicellular pathogens requiring a highly regulated cytoskeletal system for survival and pathogenicity. The functional and drug target-oriented research attention is needed for such proteins involved in pivotal signaling that are still undiscovered. This systematic review presents the identification and annotation of CRIB domain-containing proteins in unicellular eukaryotes, especially in pathogenic protozoans responsible for neglected tropical diseases and model organisms, from literature and database search. We have tried to present a crisp platform to select out proteins for targeted functional studies and drug development strategies.

## Results

The tree of life depicts that, lower eukaryotes are simple and unicellular except *Dictyostelium*, an evolutionary link between unicellular and multicellular organisms. Understanding the ancient CRIB motif in signaling effector proteins in model organism-*Dictyostelium*, and protozoan parasite-*Acanthamoeba*, *Entamoeba*, *Giardia*, *Trypanosoma* and *Leishmania* to the highest evolutionary candidate will comfort to classify them suitably for further *in vitro* and *in vivo* validation. *In silico* studies have been conducted to identify the CRIB containing repertoire of effector proteins, further cross-referenced with available literature, and finally, a new classification has also been added.

### Cdc42/Rac Interactive Binding Domain-Containing Protein in Lower Eukaryotes and Evolutionary Divergence

The complete proteome-based phylogenetic analysis of *Dictyostelium*, *Acanthamoeba*, and *Entamoeba* shows that animals and fungi are close to amoebozoa group (Song et al., 2005). Nevertheless, amoebozoa are distinct from early diverging unicellular eukaryotes, *Leishmania*, *Trypanosoma*, *Plasmodium*, *Giardia*, and plant as well. The more remarkable similarities despite their early divergence in amoebozoa and metazoan proteins translate into a generally higher degree of functional conservation between them. The universal domain architectures aid in delineating and organizing the proteins in their families and participating in particular cellular pathways in lower eukaryotes.

**FIGURE 1:**
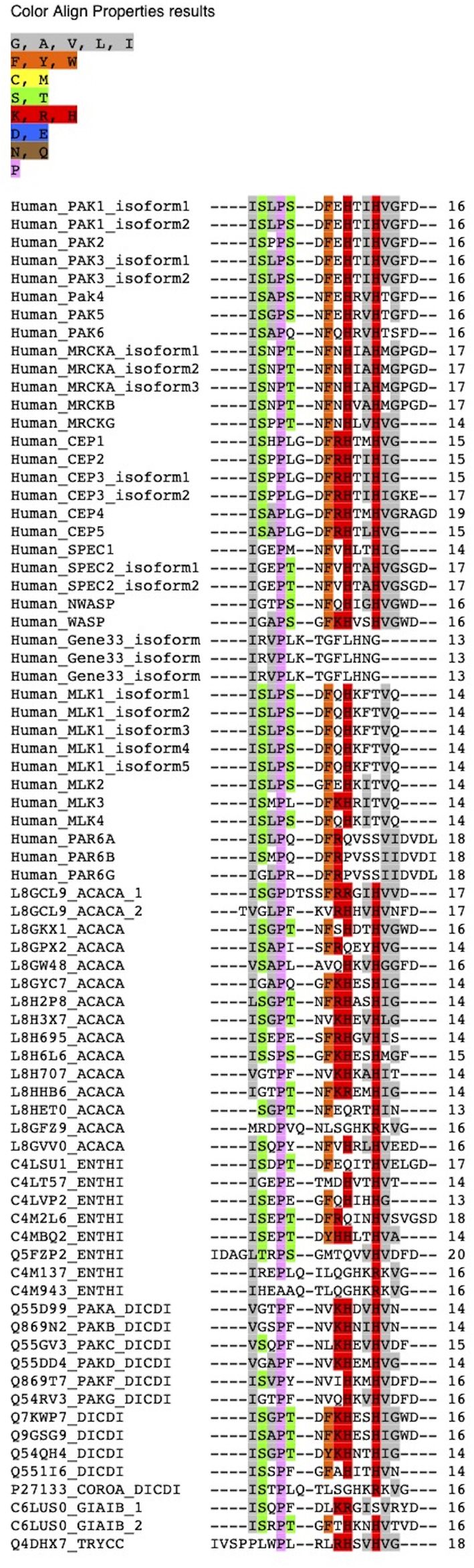
Sequence alignment of conserved CRIB motif of all the proteins of human, *Dictyostelium discoideum*, *Acanthamoeba castellani*, *Entamoeba histolytica* extracted from domain-based search from database.

The conserved sequence-based search identifies CRIB/PBD/GBD containing proteins; ten in *Dictyostelium discoideum*, fourteen in *Acanthamoeba castellani*, nine in *Entamoeba histolytica*, only one protein in *Trypanosoma cruzi*, and *Giardia lamblia*. In humans, twenty-seven CRIB containing effector proteins have been identified and reported in earlier studies (Pirone et al., 2001) (Figure 1). The available functional, biophysical and biochemical characterization has been explored in detail to classify the identified CRIB containing effector proteins. Apart from the CRIB motif, the conserved structural features in identified proteins of Amoebozoa fall into PAK or PAK like kinase and actin-associated or actin assembly protein. In *Acanthamoeba* and *Entamoeba*, very few proteins have been studied earlier, on the other hand, a lot of literature is available for *Dictyostelium* proteins. However, in other parasite protozoans (*Giardia* and *Trypanosoma*), the single identified CRIB domain protein does not show any similarities with the conserved domains of Cdc42/Rac effector. The identified CRIB containing protein families indicated here are PAK/PAK related kinase and actin assembly protein families found conventionally during evolution, while Ser/Thr and cytosolic Tyrosine kinase, adaptor family proteins present in humans are non-conventional.

### Cdc42/Rac Interactive Binding-Containing Effector Proteins in *Dictyostelium*

*Dictyostelium discoideum*, a soil-dwelling social amoeba, is a unicellular eukaryote that forms multicellular structure fruiting bodies under limiting nutrition conditions (Gaudet et al., 2008). The *D. discoideum* genome (∼34 Mb) entirely encrypts about 10,300 proteins, including numerous protein families; some are involved in fundamental processes like post-translational modification, secondary metabolism, and signal transduction belong to cellular activities like cell adhesion and cytoskeleton control (Kuspa and Loomis, 2006; Loomis, 2006). Numerous *Dictyostelium* proteins are more similar to human orthologs than yeast, probably due to higher evolutionary changes along the fungal lineage. The small, simple genome and complex transcriptome made it an easy-going prototypical organism to dissect the signaling pathways and their elements with typical relationships throughout the metazoans (Li and Purugganan, 2011; Bozzaro, 2019).

**FIGURE 2:**
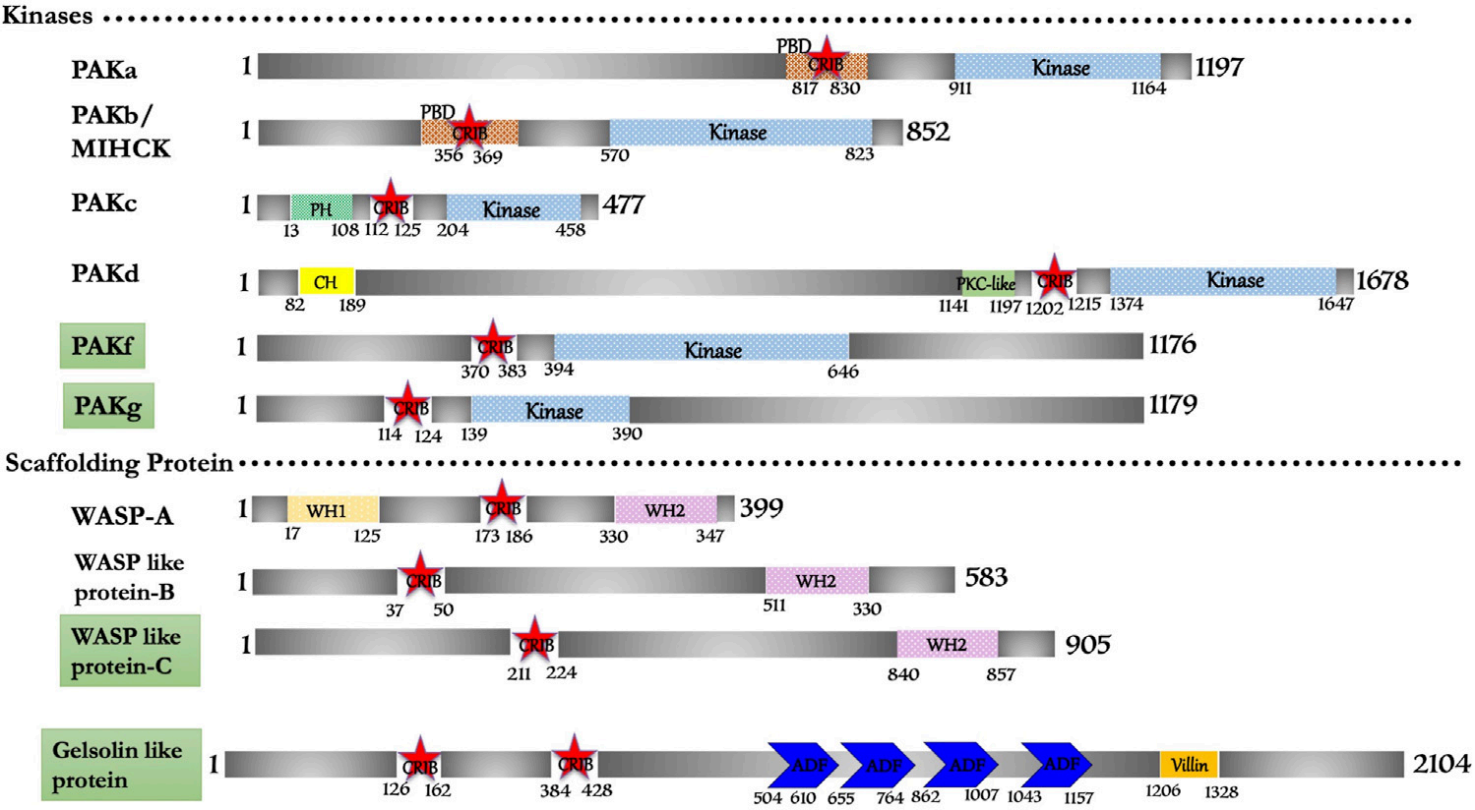
Schematic diagram of domain organization of *Dictyostelium discoideum*, CRIB domain containing proteins. The domain names and amino acid is labeled. The abbreviations for the domains are: CRIB, Cdc42/Rac interactive binding; CH, Calponin Homology; PBD, p21-activated binding domain; PH, pleckstrin-homology; WH1, Enabled/VASP homology 1; WH2, WASP homology 2; ADF, Actin depolymerizing factor. The proteins already known from the earlier literature, but not characterized yet are marked as green colored box. The proteins marked in orange (subtle effect) boxes are reported for the first time in this *in silico* study and are completely uncharacterized. Footnote: In *Dictyostelium discoideum*, PAKa-d can be studied for their protein structure and interaction studies with respective Rac repertoire or Cdc42. The functional role of PAKa-d in cell motility, adhesion, phagocytosis and chemotaxis represent their crucial role in unicellular protozoans, and comparative studies in parasite protozoans would be the scope of future research.

In this study, ten CRIB domain (or PBD/GBD) proteins were found fit functionally to be Cdc42/Rac effector proteins. Conserved sequence and structural features group them into p21-activated kinase (six protein), WASP family (three protein) and a novel gelsolin-related protein (Figure 2). However, a genomic study mentions that eight PAKs (PAKa-h) are present in *D. discoideum* (Arasada et al., 2006), with no structural and functional shreds of evidence support. In our study we found that PAKe (Q54B33) and PAKh (Q556S2) have a kinase domain but lack a consensus CRIB domain and other accessory domains present in human homologues. Thus, these two proteins may potentially be candidates of some other subfamily of Ser/Thr kinase that is a matter of further investigation or may be pseudo PAK like *E. histolytica* PAK1 (Labruyere et al., 2000; Labruyere et al., 2003). The novel gelsolin homolog identified here is encoded by the gnrC gene, categorically a putative actin-binding protein (Q551I6), which suggests its regulation by a small gtpase. This is the unique protein in *Dictyostelium* with two CRIB domains present consecutively at its N-terminal. However, no study reports any details regarding the Rac protein, which activates it.

Out of six PAK family members identified in the search, PAKa, PAKb/MIHCK, PAKc, and PAKd have some similar structural domains of human Group-I PAKs. PAKf (Q869T7) and PAKg (Q556S2) possess the conserved CRIB and kinase domain; typical structural features of PAK homologs of metazoans and yeast but need to be experimentally validated. One member has identical features to human WASP protein, while, the other two (WASP like-B (Q7KWP7) and WASP like-C (Q54QH4)) have comparable domain characteristics to human N-WASP derived as WASP-related protein (Figure 2).

In *Dictyostelium*, PAK and WASP are the CRIB-containing effectors of Rho family GTPases, which regulate chemotaxis, phagocytosis, and cytokinesis (Wilkins and Insall, 2001; Park et al., 2004). No clear homologues of Rho and Cdc42 are present, though 15 members of Rac have been reported in the Rho GTPase family (Rivero et al., 2001; Rivero and Somesh, 2002). RacB has been proposed as a functional equivalent to Cdc42 (Rivero et al., 2001). Additionally, a component of SCAR (suppressor of cAMP-receptor)/WAVE complex, four IQGAPs, ten formins, two PCH (full form) family, several lipid kinases/phospholipase and NADPH oxidase components are also present which represent CRIB-independent effectors of Rho family (Vlahou and Rivero, 2006). Experimental evidence remarkably suggested that a CRIB motif is present in coronin protein, which interacts with GDP bound gtpase (Swaminathan et al., 2014; Swaminathan et al., 2015). In general, coronin activates the actin nucleation factor Arp2/3 complex and IQGAPs (Shina and Noegel, 2008). The presence of the CRIB motif varied amongst lower and higher eukaryotes’ coronin proteins. Nevertheless, the sequence comparison of the conserved CRIB motif and the coronin CRIB region indicated moderate similarity while displaying Cdc42/Rac binding. Reasonable similarity can possibly explain that the region not annotated as CRIB in protein domain databases can contribute to the protein-protein interaction mechanism.

WASP family consist of one WASP, two WASP related and one WASP like (SCAR) subfamilies proteins, which are the positive regulators of Arp2/3 complex in actin polymerization (Seastone et al., 2001; Myers et al., 2005). WASP controls Arp2/3 complex spatially and temporally in *D. discoideum* via interaction of Rac with the CRIB motif. WASP, an actin nucleation-promoting factor, also functions as a controller of cellular localization of Rac, contributing to the maintenance of front-rear polarity (Amato et al., 2019). WASP-A encoded by wasA gene has a WH1 (WASP-homology 1) domain that interacts with poly-Proline helices (Myers et al., 2005). RacC works as a connection between WASP activation and chemo-attractant stimulation in the signaling pathway regulating F-actin assembly during chemotaxis (Han et al., 2006). WASP-B regulates F-actin polymerization through attenuation that is important for regulating the dynamics of pseudopod extension and retraction (Chung et al., 2013). However, SCAR subfamily proteins possess a C-terminal VCA domain akin to human WASP and N-WASP but lack an extended N-terminal WH1 region and GBD domains (Symons et al., 1996). The human homolog of SCAR is called WAVE. SCAR/WAVE is a multi-protein complex with PIR121, Nap125, Abi2, and HSp300 components, each encoded by a single gene present in the *Dictyostelium* genome (Blagg et al., 2003). WASP compensates for the loss of function of SCAR/WAVE proteins in *Dictyostelium* (Veltman et al., 2012).

PAK family has six proteins positioned in two different clades, classified in two separate classes; PAKa-d and PAKf/g, based on phylogenetic analysis of the catalytic kinase domain. The consequence of such distinction is not clear in *Dictyostelium* to humans who also have two PAK groups (Arasada et al., 2006). The class I PAKs in *Dictyostelium* functionally established to be involved in cell polarity, actin-myosin assemble and phagocytosis (Lee et al., 2004; Yang et al., 2013; Garcia et al., 2014; Phillips and Gomer, 2014). The six PAK isoforms share high sequence identity ∼50–70% in the catalytic kinase domain and PBD regions, while the rarely display any homology outside these regions. PAKa and PAKb, both of which have CRIB/AI domains that have been linked to myosin II regulation (de la Roche and Cote, 2001).

*Dictyostelium* PAKa possesses a potential poly-Proline tract for SH3 domain interaction, a highly acidic N-terminal domain followed by a CRIB and a C-terminal kinase domain. The CRIB/PBD domain preferentially interacts with DdRac1B and HsCdc42 and then translocate to myosin II filament to regulate myosin heavy chain kinases (MHCKs). PAKa inhibits MHCK-B, C, and D to stabilize myosin II assembly in response to upstream cAMP response. Despite dynamic subcellular localization, PAKa co-localizes with myosin in all the cell movement process (de la Roche and Cote, 2001). PAKa was also identified at the cytokinesis cleavage furrow, and localized to rear end of the polarized migrating cell, and posterior cortex during chemotaxis (Chung and Firtel, 1999; Muller-Taubenberger et al., 2002). The signaling cascades regulated by PAKa are also dependent on coronin (Swaminathan et al., 2015).

*Dictyostelium* PAKb consists of an N-terminal Proline-rich region, followed by a consensus PBD, a Glutamine and Asparagine residues rich linker, and a C-terminal kinase domain for catalysis. DdPAKb, previously termed as myosin I heavy chain kinase (MIHCK), was identified (Lee et al., 1996) through its phosphorylation and regulation of a single-headed myosin I (DdMyoD) (de la Roche and Cote, 2001). PAKb phosphorylates TEDS (Thr, Glu, Asp, or Ser residue) rule site located in the MyoD motor (de la Roche and Cote, 2001). *Dictyostelium* Rac1a/b/c, RacA (a RhoBTB protein), RacB, RacC and RacF1 activate PAKb to regulate myosin driven motility on actin. PAKb localizes in the cytosol and enriches at the leading edge of the cells during migration, macropinocytosis, and phagocytosis, sites that show prevalence of myosin I as well (de la Roche et al., 2005). Thus PAKb plays pivotal role in myosin I activation during these events. However, loss of function mutants’ have impaired functions dependent on myosin I (Wu et al., 1996). In contrast, constitutively active PAKb mutant increases the rate of myosin I dependent processes such as pinocytosis/phagocytosis and disrupts cytokinesis. The constitutively active and C-terminal truncated active PAKb shows localization at rear-end of the migrating cell and cleavage-furrow during cell division (Yang et al., 2013). The notable fact here is the opposite localization of PAKa and PAKb in migrating cells, one at the rear end and the other at the posterior end, respectively, suggests that the two proteins do not have an overlapping function. However, both PAKa and PAKb function synergistically during phagocytosis and not pinocytosis.

The structural features of PAKc include a PH domain followed by a CRIB and a kinase domain, with a C-terminal extension G_ßγ_ binding domain. PAKc PH domain, related to the fungal Cla4p-like PAKs, is alone responsible for the cytosolic localization (Phillips and Gomer, 2014). The PH plus CRIB domain exhibits weak membrane localization in response to chemo-attractant stimulation (Lee et al., 2004). PAKc is activated rapidly and transiently in response to chemo-attractant stimulation that enriches it at the plasma membrane. PAKc functions to inhibit lateral pseudopodia to restrict pseudopod formation to the plasma membrane facing the chemo-attractant source (Lee et al., 2004). PAKc CRIB domain preferentially binds to RacC GTP-bound form. The highly conserved Arginine 34 is required for inositol binding in the PH domain. The CRIB domain is strongly similar to human PAK1 consisting of overlapping CRIB and AID (auto-inhibitory domain). The C-terminal G_ßγ_ binding domain shows strong conservation of the C-terminal yeast Ste20, required for transient localization to the *Dictyostelium* plasma membrane (Lee et al., 2004).

PAKd contains an N-terminal CH domain and additional C1 domain upstream of the CRIB domain. PAKd was implicated in F-actin aggregation during developmental processes, and actin polymerization in response to stimulation by a chemo attractant (Phillips and Gomer, 2014). Upon cell starvation, PAKd moves to cellular extensions, suggesting its presence in the Golgi apparatus (Garcia et al., 2014). PAKd kinase activity is regulated through the binding of CRIB domain to activated Cdc42/Rac molecules.

Experimental records strongly confirm that *Dictyostelium* coronins (Coronin A and B) have a Rac activated CRIB motif (Uetrecht and Bear, 2006). None of the previous studies on CRIB containing proteins included coronin family under Cdc42/Rac effector proteins (Clemen et al., 2008). In *D. discoideum* CoroninA, residues 117–133 harbor the CRIB motif highly homologous to CRIB motifs of other conventional effector proteins. Structural characterization indicates that half of the CRIB motif lies on the solvent accessible face, while the other half is embedded inside. Coronin prefers the GDP form of GTPases for binding to the CRIB motif, which is interestingly exceptional to all other CRIB containing proteins. Analysis of coronin lacking mutants reveals its role in cell motility, phagocytosis and cytokinesis (Vinet et al., 2014). Coronin functions as a Rho protein GDP dissociation inhibitor (RhoGDI) that interacts with Rac GTPases in their inactive GDP bound form, thus preventing their availability to PAKs. It also interacts with PAKs (PAKa) directly to regulate their activity (Swaminathan et al., 2015).

### Cdc42/Rac Interactive Binding-Containing Effector Proteins in *Acanthamoeba*

*Acanthamoeba* is the solitary free-living soil amoeba that diverged earlier than other amoebozoans (Shabardina et al., 2018; Corsaro, 2020). The genome contains ∼15,455 coding genes and comparative genomic studies from other metazoans established many putative protein families who play an interpreting role of cytoskeleton machinery and signaling related to cell motility and cytokinesis (Clarke et al., 2013). The putative proteins involved in cytoskeleton regulation through small GTPases in downstream pathways are not explored thoroughly (Mullins and Pollard, 1999).

**FIGURE 3:**
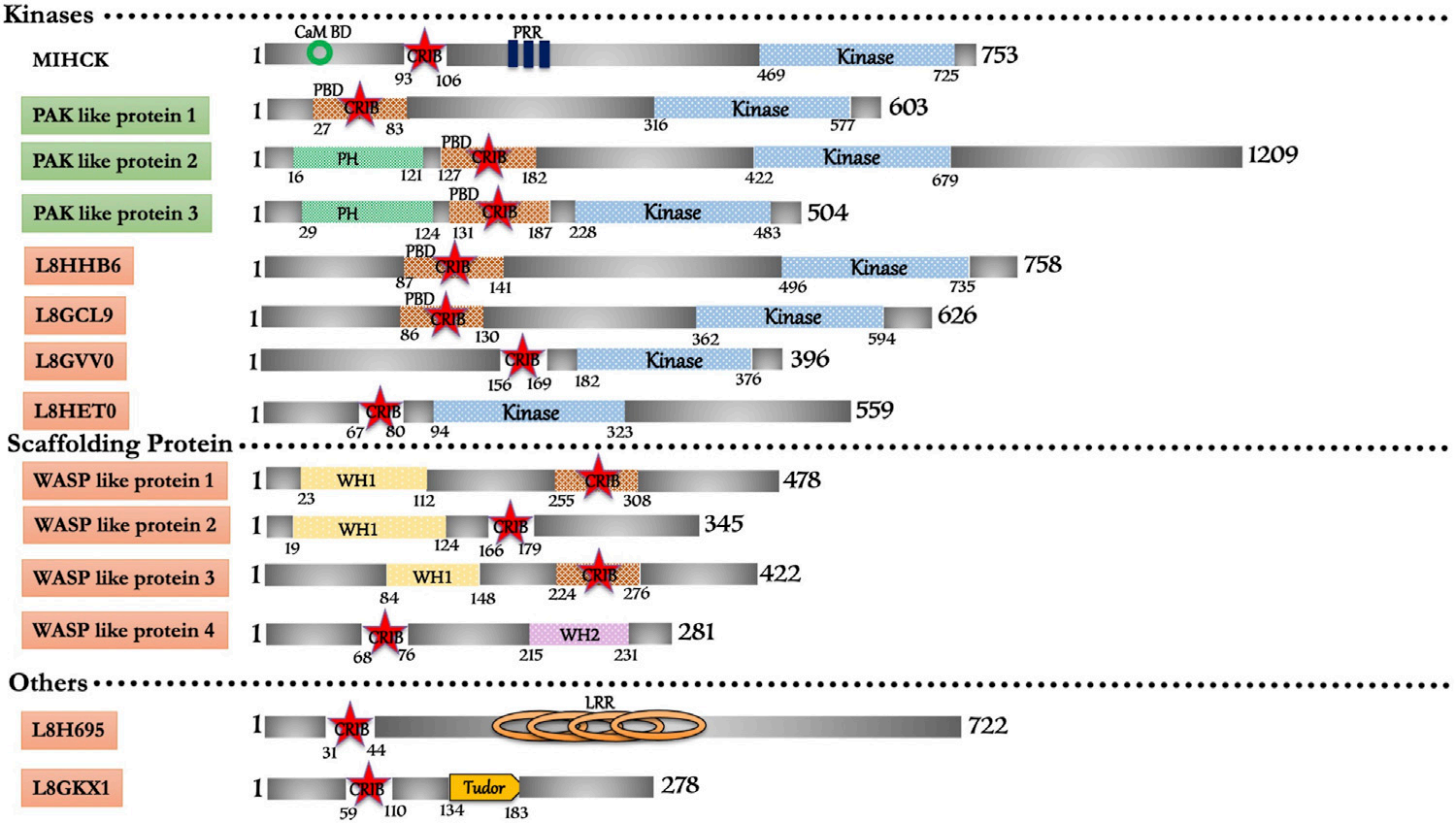
Schematic diagram of domain organization of *Acanthamoeba castellani* CRIB domain containing proteins. The abbreviations for the domains are: CRIB, Cdc42/Rac interactive binding; CaM BD, Calmodulin Binding Domain; LRR, Leucine Rich Repeat; PBD, p21-activated binding domain PH, pleckstrin-homology; PRR, Proline Rich Region; WH1, Enabled/VASP homology 1; WH2, WASP homology 2. The proteins already known from the earlier literature, but not characterized yet are marked as green colored box. The proteins marked in orange (subtle effect) boxes are reported for the first time in this *in silico* study and are completely uncharacterized. Footnote: In Acanthamoeba castellani, the biochemical characterization is only available for MIHCK, reported 2 decades ago. However, in our study, we report the highest number of CRIB domain-containing proteins from this organism. Their identification opens all kinds of structural, biochemical, biophysical and cellular study avenues for further research. The detailed study on the proteins with entirely novel domain combinations is highly relevant for further research as drug candidates.

*In silico* search for the conserved CRIB domain retrieved fourteen proteins in *Acanthamoeba castellanii* (Figure 3). The only characterized protein in this repertoire is a Myosin I heavy chain kinase (MIHCK), belongs to the p21 activated kinase family (Brzeska et al., 1997). Including MIHCK (Q93107), seven proteins (L8GCL9, L8GPX2, L8GVV0, L8GW48, L8H707, L8HHB6, and L8HET0) have kinase domains homologous to human PAKs along with some variant domains. L8GPX2 shares a domain with myosin I heavy chain kinase, while, L8H707 and L8GW48 have substantial similarities with PAK proteins. L8HHB6 (39%) and L8GCL9 (42%) are potential Ser/Thr kinases that show moderate similarity with human PAK6 and can be categorized under the PAK family after experimental validation. L8HET0 kinase domain shows 24% similarity with HsMRCKβ but no other domains so it could be probable PAK family member. L8GVV0 shows 30% similarity with HsPAK3 can also be prospective PAK family member subjected to experimental backing. Two proteins (L8H2P8 and L8H6L6) with WH1 domain and strong homology with WASP proteins could be potential WASP proteins in *Acanthamoeba*. Apart from these two, another protein (L8H3X7) possesses WASP like domain characteristics/homology with WASP related proteins of metazoans. Two proteins (L8GKX1 and L8GYC7) composed of unique domains along with the CRIB domain, which does not have homology with any conventional/non-conventional proteins of higher eukaryotes, can be classified as p21 rho-binding domain-containing protein. We also found one protein (L8H695) with Leucine-rich repeats along with the CRIB domain, and have classified it as a unique group. Lastly, L8GFZ9 was with similar structural features to the coronin family, but the functional role and interactive GTPase molecule are yet to be explored. The extensive repertoire of CRIB containing proteins present in *Acanthamoeba* indicates and supports the ancient origin of the CRIB motif and its linkage to various effector proteins of Rho mediated signaling (see Supplementary Table S1). However, the relation with the Rho family of protein and detailed functional study will help link these proteins with cellular processes and their subcellular localization.

*Acanthamoeba* MIHCK, member of the PAK family, phosphorylates a heavy chain of myosin IC and activates it. MIHCK, from *Dictyostelium*, was validated as PAKb, which performs a similar function to phosphorylate and activate myosin I. AcMIHCK has a homologous region to human PAK1, PBD (Residue 93–149), which includes the CRIB motif (Residue 93–100) and as IS domain, but lacks the kinase inhibitory (KI) domain region. It also has a putative calmodulin-binding region at its N-terminal, before PBD (Brzeska et al., 2001). The typical C-terminal Ser/Thr kinase domain has the characteristic Ser-627 phosphorylation site. The region between PBD and catalytic domain (residue 158–449) is highly Proline-rich, including multiple PXXP motifs (class I) that provide potential binding to SH3 domains (Brzeska et al., 1996). *Acanthamoeba* MIHCK mechanism of regulation is quite similar to mammalian PAK1. *Acanhtamoeba* MIHCK is fully phosphorylated (Brzeska et al., 1990a; Brzeska et al., 1999), while *Dictyostelium* MIHCK is partially activated *in vitro* by autophosphorylation in the presence of Rac and lipids (Lee et al., 1998). Human PAK1 also requires Rac or lipids for autophosphorylation (Manser et al., 1994). Calcium-dependent calmodulin inhibits phospholipids’ stimulation in *Acanthamoeba* MIHCK (Brzeska et al., 1992) and *Dictyostelium* MIHCK/PAKb (Lee et al., 1998) but is not required for mammalian PAKs. Also, the lipid that activates mammalian PAK1 (Bokoch et al., 1998) differs from those that activate *Acanthamoeba* and *Dictyostelium* proteins (Brzeska et al., 1990a; Brzeska et al., 1990b).

### Cdc42/Rac Interactive Binding-Containing Effector Proteins in *Entamoeba*

*Entamoeba histolytica* is a primitive unicellular eukaryote and amitrochondrian protozoan parasite, which causes dysentery and liver abscess. Amoebic pathogenicity is selected coincidently in the lumen of the intestine because the parasite uses the same methods to kill bacteria or cause disease by damaging the host cells (Bosch and Siderovski, 2015). Amoebic phagocytosis and its mechanism show similarities with the action of macrophages during the phagocytosis of bacteria and unwanted cells, which supports the idea of coincidental selection (Ghosh and Samuelson, 1997; Labruyere et al., 2019). The parasite uses anterior pseudopods and posterior uroids to move inside the human intestine. The host complement system, lectin ConA (multivalent), and anti-amoeba antibodies target the invading amoebic cell, initiating the formation of cap by rearward recruitment of surface receptors and increasing the local receptor-ligand concentration (Calderon, 1980). The defense mechanism of *E. histolytica* against host immune response includes surface receptor capping in the uroids and membrane shedding (Espinosa-Cantellano and Martinez-Palomo, 1994). *E. histolytica* cytoskeleton functions actively in the capping process (Espinosa-Cantellano and Martinez-Palomo, 1994; Rath and Gourinath, 2020). The invasion and survival inside the host tissue are maintained through the phagocytosis of RBC, lumen cells and surrounding cells. It has been demonstrated the role of cytoskeleton assembly in the *Entamoeba* and a human macrophage (Marchat et al., 2020). Phagocytosis is a dynamic and regulated process that involved a varsity of proteins ranging from actin-binding protein, motor protein, small GTPases, kinases and phosphatases (Godbold and Mann, 1998; Marquay Markiewicz et al., 2011; Anwar and Gourinath, 2016; Gautam et al., 2017; Agarwal et al., 2019; Gautam et al., 2019). ∼20 Rho family GTPases and numerous downstream signaling effectors are present in these single-celled trophozoites that coordinate actin dynamics in pathogenesis-related processes. Hence, cell migration and chemotaxis, followed by adherence to the epithelium in the host intestine, and host cell killing and phagocytosis are all regulated by Rho family signaling toolkit (Bosch and Siderovski, 2013).

**FIGURE 4:**
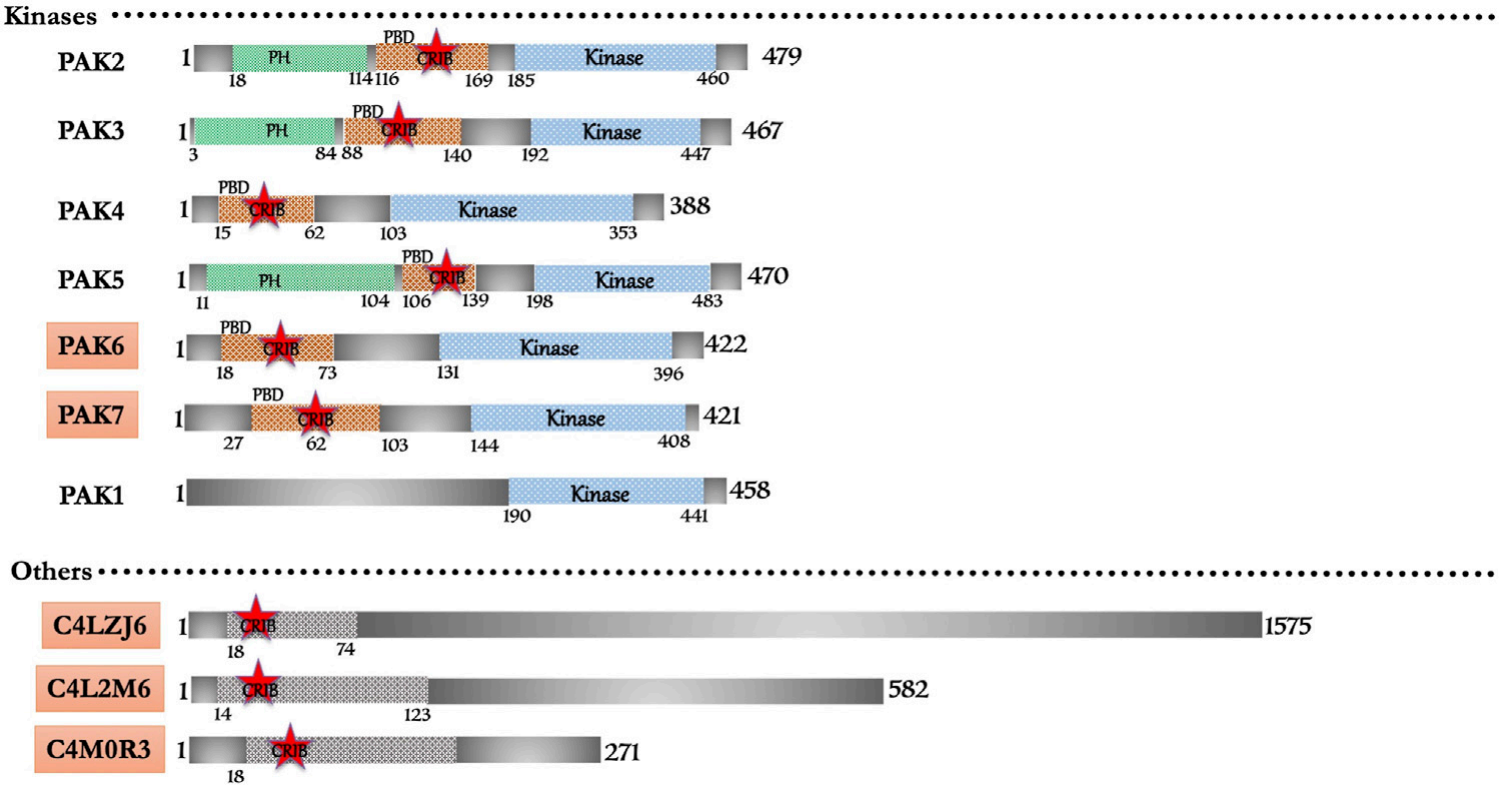
Schematic diagram of domain organization of *Entamoeba histolytica* CRIB domain containing proteins. The abbreviations for the domains are: CRIB, Cdc42/Rac interactive binding; PBD, p21-activated binding domain PH, pleckstrin-homology. The proteins already known from the earlier literature, but not characterized yet are marked as green colored box. The proteins marked in orange (subtle effect) boxes are reported for the first time in this *in silico* study and are completely uncharacterized. Footnote: In Entamoeba histolytica, PAK1 and PAK2 have been reported as crucial kinases in the survival of this pathogen and are intricately involved in the phagocytic process. PAK3 and PAK4 characterization also showed a promising role in pathogenesis. Structural and biophysical characterization is the prospective future of the already recognized proteins apart from the ones identified in this study. Experimental research on these proteins will lead to new potential drugs targets against the single drug (metronidazole) available in the market. The development of new drugs is necessary due to the reports of drug resistance found in laboratory cultured amoebic cells.

The domain-based search retrieved a total of nine CRIB domain-containing proteins (Figure 4). The homology with metazoans proteins classifies them under conventional effector proteins of Rac/Cdc42. Six (PAK2-7) and one pseudo-PAK (PAK1) belong to the p21-activated kinase with their typical PBD and kinase domain architecture. However, an earlier study shows that one isoform of PAK lacks CRIB/PBD domain in N-terminal but has C-terminal kinase domain homologous to yeast ste20 (Gangopadhyay et al., 1997). Earlier kinome study (Anamika et al., 2008) predicted 17 homologous PAKs in the *E. histolytica* genome. Only with the six homologues match the typical PAK features, and thus, it is now confirmed that *Entamoeba* possesses only six PAK isoforms, including one pseudo-PAK. Interestingly, out of six, three PAKs (PAK2, PAK3 and PAK5) have additional PH domains (Figure 4). Three proteins (C4M2L6, C4LZJ6, C4M0R3) have a domain that is homologous to C-terminal of human WASP, but no experimental studies have so far been performed to characterize its structure and function (Figure 4). The Arp2/3 complex nucleates new actin filaments, when activated by nucleation promoting factors like WASP or SCAR. No available research yet describes any WASP or SCAR protein in *Entamoeba*, hence, the actin nucleation activity may be regulated by other unidentified proteins. Exploring the predicted putative WASP here might reveal its role in actin nucleation and regulation of actin cytoskeleton. However, CARMIL protein binds Arp2/3 complex with an exact mechanism used by WASP via its acidic motif. CARMIL homologues have been discovered through proteomic analysis of the phagosome (Okada et al., 2005; Clark et al., 2007; Tolstrup et al., 2007) and they further provide essential clues for understanding actin nucleation. A recent *in silico* study on actin-binding proteins (Rath and Gourinath, 2020) from our lab shows that *E. histolytica* harbors three WASH (Wiskoit-Aldrich syndrome protein and SCAR homolog) proteins C4MBT4, C4LTV1 and C4M2Y0. Additionally, *E. histolytica* encodes six formin genes that accelerate actin filament assembly in eukaryotic cells, however, no IQGAPs are reported yet. Three proteins (C4M137, C4M943, C4M5U0), which have similar structural features to the coronin protein family, are also present in *Entamoeba* (see Supplementary Table S1) but the CRIB motif is not yet defined it them like *Dictyostelium*. Out of the seven PAK family members, the detailed account of experimental evidence is available only for five; PAK6 and PAK7 have not been characterized yet (see Supplementary Table S1).

*Entamoeba* PAK (PAK1) is a pseudo-PAK because it lacks the consensus CRIB motif (or PBD); still, it interacts with EhRac1 via the N-terminal region, suggesting that it is a regulatory domain necessary for the maintenance of cell polarity (Labruyere et al., 2003). Migrating trophozoites display PAK1 at their leading edge, where it functions in amoeboid cellular polarity and motility, along with human red blood cell phagocytosis (Labruyere et al., 2000). The protein shares 33% identity with rat KPAK and yeast STE20 (Gangopadhyay et al., 1997). The Proline-rich N-terminal domain can potentially bind to SH3 domains of adapters like Nck (non-catalytic region of Tyrosine kinase adapter protein) or PIX (PAK Interacting eXchange factor) (Galisteo et al., 1996; Manser et al., 1998). Constitutive EhPAK expression alters the new adhesion site formation in *E. histolytica* (Labruyere et al., 2003).

PAK2 PBD selectively binds activated EhRacA during receptor capping and collagen matrix invasion (Arias-Romero et al., 2006). PAK2 probably controls cell movement, surface receptor capping and cytokinesis (Arias-Romero et al., 2006). The biochemical studies conducted on the C-terminal kinase domain of PAK2 described its activity towards myelin basic protein. Interestingly, the PAK and EhRacA complex homology model showed the specific interaction in PAK2 residues Met-121 and His-123 with RacA Tyr-40; and PAK2 residue Phe-145 with RacA Asp-63, Arg-66, Leu-67 and Leu-70 (Arias-Romero et al., 2006). A detailed investigation to elucidate the critical residues influencing the binding energy would guide the rationale development of small molecules that inhibit such interaction. The possible interaction of other GTPases with PAK2 can also be investigated experimentally.

Un-stimulated cells show cytoplasmic PAK3 distribution, while capping protein induction relocates it to the caps (Dutta et al., 2007). PAK3 undergoes autophosphorylation and phosphorylates histone H1 *in vivo*, and *in vitro* studies displays kinase activity in the absence of small GTPases (Dutta et al., 2007). Maximum enzymatic activity is achieved after the autophosphorylation of a critical residue present in the activation-loop of many protein kinases. It is yet to be established whether an increase in activity or change in localization is observed upon gtpase binding. PAK3 sequential feature reports a PH domain (residues 2–82), a PBD/CRIB domain (residues 84–141) and a kinase domain (residue 142–447) at its C-terminal. PAK3 shares 40% identity (50% similarity) with *Dictyostelium* PAK (PAKc). All the typically conserved XI subdomains from Ser/Thr kinases are visible in its kinase domain. The only variation observed, is the replacement of the conserved Leucine by Tyrosine in subdomain VII.

PAK4 and PAK5 are highly specific effectors of EhRacC (Bosch and Siderovski, 2015). EhRacC^Q65L^GTP and EhPAK4-PBD reveal a deviation of PBD α-helix in an otherwise conserved Rho/effector interface. The side chains of EhPAK4 PBD residues line up the EhRacC binding interface. The similar residues are well conserved in EhPAK5, hinting at a common interaction surface for the same Rho gtpase. Asp-17 of EhPAK4 (Glu-108 in EhPAK5) forms a salt bridge with Arg-30 of EhRacC, while Phe-21 of EhPAK4 (Tyr-112 of EhPAK5) contributes towards a hydrophobic RacC interface (Bosch and Siderovski, 2015). Further experiments need to be conducted to for RacC effectors PAK4 and PAK5 to elucidate their biological functions. We still lack the knowledge that correlates the autoinhibition modes of *E. histolytica* PAK isoforms with mammalian group-I and group-II PAKs. Here, we also acknowledge the unresolved question of the signaling specificity between Rho family gtpase and PAKs.

*Entamoeba* has three coronins; two belonging to short tail (Coronin1: C4M943, Coronin 2: C4M137) are 70% identical to each other, and one long tail (Coronin 3: C4M5U0). The predicted function of long coronin is crucial in the amoeboid migration and pseudopod regulation (Tolstrup et al., 2007).

### Cdc42/Rac Interactive Binding-Containing Effector Proteins in *Giardia*

The genus *Giardia* comprises several species that inhabit intestinal tracts of vertebrates (fish, amphibians, reptiles, birds, rodents). It is one of the most pervasive intestinal pathogens that infects a wide range of mammals; for example, human and agricultural livestock such as cattle and sheep. However, *Giardia lamblia* (alternatively referred to as *Giardia intestinalis* and *Giardia duodenalis*) infects and causes giardiasis in humans, suggesting a zoonotic transmission (Ryan and Caccio, 2013). The life cycle is simple, involving two morphogenetic stages; 1) cyst form, which is environmentally resistant and infectious, and 2) vegetative trophozoite stage, which colonizes the small intestine and becomes invasive to cause disease. During encystation, the parasite relays the signals to produce, transport, and secrete the cyst wall protein (CWP). It has been demonstrated that flagella and disk structures modulate motility and host intestinal epithelial cell attachment (Di Genova and Tonelli, 2016). The molecular mechanism behind the regulation of these processes remains abstract. The sole Rho family gtpase, GlRac, regulates endomembrane organization and CWP trafficking (Krtkova et al., 2016). Subcellular localization studies indicate the association of GlRac with endoplasmic reticulum and Golgi apparatus like encystation-specific vesicles (ESV).

The CRIB domain search identifies only one protein (C6LUS0), which has the CRIB and the kinase domains homologous with other PAKs (see Supplementary Table S1). The earlier studies indicated that *Giardia lamblia* has only one Rac in the complete Rho family, so the identified protein in our search can be the prospective effector protein (Krtkova et al., 2016). The interaction and co-localization studies on proposed protein with known Rac will provide thoughtful insights on the signaling mechanism of cytoskeleton assembly in the parasite.

### Cdc42/Rac Interactive Binding-Containing Effector Proteins in *Trypanosoma*

*Trypanosoma cruzi* causes an encumbering severe illness in humans, known as the Chagas disease, which affects millions of people globally (de Souza et al., 2010). The various species of *Trypanosoma* belongs to kinetoplastid protozoans in an evolutionary context (Gupta et al., 2020). The complex life cycle involves four developmental stages: 1) epimastigotes; 2) metacyclic trypomastigotes; 3) amastigotes; and 4) bloodstream trypomastigotes (Zuma et al., 2021). Trypomastigotes and extracellular amastigotes are the only infective forms that are able to invade almost any nucleated host cell (Ferri and Edreira, 2021). During the invasion, bidirectional signaling pathways are triggered in both the parasite and the host cell. The CRIB domain search identifies only one protein (Q4DHX7) in *Trypanosoma cruzi* (see Supplementary Table S1) which intrigued us to carryout in depth literature search for cytoskeletal regulation signaling pathways. However, we cannot categorize this CRIB domain-containing protein in any existing families of effector protein because it doesn’t any depict homologous features to other eukaryotic proteins. Further investigation is required to know more about the protein function and relation to the CRIB domain-containing protein family from other lower eukaryotes.

Interestingly, few proteins have been found that regulate cytoskeletal pathways but are only related to the invasion of the extracellular amastigote form of *T. Cruzi*. Extracellular amastigote engulfed by mammalian cells via phagocytic cup based on actin-dependent cytoskeleton changes (Mortara et al., 2005). Extracellular amastigotes secretes proteins like P21, mevalonate kinase (MVK) and specific-surface protein 4 (Ssp4), which mediate host cell signaling during phagocytosis. P21 is a 21 kDa secretory protein (da Silva et al., 2009), related to ERK and PI3K signaling pathways during phagocytosis and cytoskeleton remodelling (Rodrigues et al., 2012; Teixeira et al., 2015). The recombinant version of P21 (rP21) interacts with the CXCR4 chemokine receptor, inducing actin assembly to drive phagocytosis and modulate the PI3K-dependent expression of an actin-related gene (da Silva et al., 2009; Rodrigues et al., 2012). TcMVK is involved in protein glycosylation and cytoskeletal assembly through activation of p38/ERK, FAK (focal adhesion kinase) components and PAK signaling trails (Ferreira et al., 2016). Amastigote form invades the host cell through Ssp-4, another secretory molecule predicted to function as Rac1/WAVE2 and Cdc42/N-WASP signaling mediators (Florentino et al., 2018). TcSsp4 is majorly a surface GPI-anchored glycoprotein whose expression doesn’t correlate with infection, but glycosylation of protein is linked with host cell invasion. Highly infective strain’s amastigotes secrete a differentially glycosylated Ssp4 that recruits Galectin-3 (Gal3) to mediate host cell surface and parasite interaction (Florentino et al., 2018). Recent studies on actin-binding proteins from kinetoplastids, proposed a protein (Q4DEX0) as coronin because it has homologous WD repeat and coronin domains characteristic to amoebozoa and higher eukaryotes (Gupta et al., 2020).

### Cdc42/Rac Interactive Binding-Containing Effector Proteins in *Leishmania*

*Leishmania donovani* also belongs to kinetoplastids, a unicellular protozoan parasite causing a fatal disease, visceral leishmaniasis, in humans. In vertebrates, it is present as invasive promastigote and amastigote, which cause infection. The best survival strategy used by promastigotes during establishment of infection in macrophages is to inhibit the fusion of phagosome and endosome (Desjardins and Descoteaux, 1997). Lipophosphoglycan (LPG), a significant surface glycoconjugate of promastigote, is crucial for intracellular survival (Handman et al., 1986). The pathogen uses the human host macrophage cell cytoskeletal assembly to sustain and prevent phagosome maturation (Scianimanico et al., 1999; Lodge and Descoteaux, 2008). However, recent report on the actin-binding protein repertoire is classifying the presence of various proteins in the pathogen itself (Gupta et al., 2020). Earlier information reveals that *L. donovani* recruits human Cdc42 and Rac1 to form an F-actin coat around its phagosome as protective measure from macrophage killing (Lodge and Descoteaux, 2005; Lerm et al., 2006).

The domain search retrieves one protein (Q4QEZ0), which has conserved Ser/Thr kinase domain homologous to PAK catalytic domain but no CRIB domain was present in it (see Supplementary Table S1). A coronin homolog protein (E9BGF4) is reported recently in the genome-based studies (Gupta et al., 2020). The functional characterization to link it with CRIB containing effector proteins is yet to be established.

### Cdc42/Rac Interactive Binding-Containing Effector Proteins in the Human Host

**FIGURE 5:**
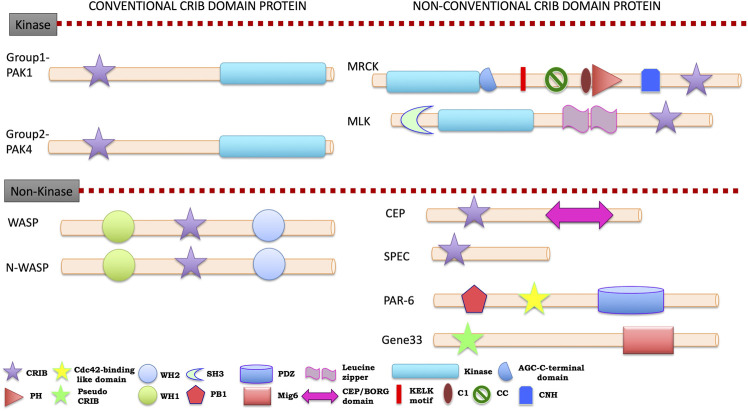
Cartoon diagram of signature domain organization of human CRIB domain containing protein families A colour key to the domain names and symbols is given below of classification. The abbreviations for the domains are: CRIB, Cdc42/Rac interactive binding; PH, pleckstrin-homology; WH1, Enabled/VASP homology 1; WH2, WASP homology 2; SH3, Src-homology 3 doamin; PB1, Phox and Bem1; PDZ, PSD-95, *Drosophila* discs large, and the adherens junction protein, ZO 1; CEP, CRIB effector protein; CNH, Citron homology domain. The proteins divided into conventional and unconventional classification on the basis of their evolutionary link with unicellular eukaryotes.

Human is placed as the top ranking evolutionary evolved organism, but it is a host for several pathogens. The understanding of molecular mechanisms of host and pathogen proteins helps to prevent infections and diseases. The cellular signaling process is complex in this system due to presence of multi-layered system of tissues and organs. However, cytoskeleton signaling and its regulation are somehow similar in most of the cells and different in some cells at the same time. Investigations on complexity in regulating cytoskeleton dynamics, mediated by Rho family GTPases is fundamental to processes like motility (Murali and Rajalingam, 2014), adhesion, differentiation and development. All the twenty-seven proteins, which have been identified in the CRIB domain-based search, are effector proteins of Rac/Cdc42. The brief information of CRIB containing effectors is accounted in this study to understand the link and origin of CRIB motif and association with protein in social amoebas (Kumar et al., 2009), intestinal pathogens and kinetoplastids. In the human proteome, nine different CRIB containing protein families are present (Figure 5, see Supplementary Table S2). Additionally, six coronin isoforms are also identified in human; some of them have shown the presence of CRIB motif via experimental evidence while others need to be explored (see Supplementary Table S2).

#### p21-Activated Kinases

Human PAKs are thoroughly studied, including the elucidation of their structure, function, localization and regulatory mechanism in cells. PAK isoforms have been categorized in Group-I (PAK1, 2, 3) and Group-II (PAK4, 5, 6). Most of the PAKs are ubiquitously expressed, but some are restricted to tissue specific expression (Murali and Rajalingam, 2014). PAK phosphorylates numerous substrates (membrane, cytosolic, mitochondria and nuclear) and act by remodeling cytoskeleton, employing scaffold and shuttling to specific subcellular compartment (Lian et al., 2002; Kumar et al., 2017). It has been clearly understood from evidences that their dysregulation leads to disruption of cellular homeostasis and severely impacts key cellular functions (Bokoch, 2003). Some PAKs are associated with numerous defects and disease (Chan and Manser, 2012) majorly, cancer (Kumar et al., 2006), neurological (Ma et al., 2012), and cardiac disorders (Ke et al., 2014). The in depth functional details can be referred from the recent reviews (Zhao and Manser, 2005; Rane and Minden, 2014; Kumar et al., 2017), while this study presents the classification details based of domain architecture and structural features. Both the PAK groups possess a PBD at N-terminus, an auto inhibitory domain (AID), and a kinase domain at C-terminus. The PBD domain is similar in both groups whereas AID domain of Group-I is partly similar to group-II with minor modifications. The regulatory kinase domain is structurally different and shows distinct activation mechanism in both groups (Ha et al., 2015). The kinase activity of group-I PAKs is initiated in the presence of Rac/Cdc42, while group-II doesn’t require Rac/Cdc42 stimulation for its constitutive activity. Many publications (Baker et al., 2014; Rane and Minden, 2014) highlight that group-I activity modifies through two PAK molecules acting as a dimer to exert a reciprocal auto inhibitory activity (Buchwald et al., 2001). Group-I PAKs are also stimulated by interaction of its PIX motif (PXXP motif at Proline-rich region) present between PBD and kinase domain with SH3 domain in signaling molecule, phosphorylation by 3-phospho-insositide dependent kinase1, AKT and JAK, and binding of phospholipids, exchange factor β-PIX or SH3 proteins such as NCK1 and GRB2 (summary by (Kumar et al., 2017)). The emerging role of PAK1 as potential therapeutic target in cancer was recently reviewed (Rane and Minden, 2019; Yao et al., 2020) comprehensively, elaborating interesting facts and classical evidences to understand its role in many oncogenic signaling pathways (Senapedis et al., 2016; Yao et al., 2020).

#### Wiskott-Aldrich Syndrome Proteins

The 2 decades of extensive evidences suggest that WASP family proteins have widened into five subfamilies in vertebrates including humans: 1) WASP and neural-WASP (N-WASP; also known as WASL), 2) three WASP family Verprolin homologue isoforms (WAVE1-3; also known as SCAR1-3 or WASF1-3), 3) WASP homolog associated with actin, membranes and microtubules (WHAMM), WASP and SCAR homologue (WASH; also known as WASHC1), and 4) junction-mediating regulatory protein (JMY) (Alekhina et al., 2017). WASP protein participates in innate and adaptive immune response through regulation of actin cytoskeleton-dependent cellular processes, including immune synapse formation, cell signaling, migration and cytokine release (Thrasher and Burns, 2010; Rivers and Thrasher, 2017). Most of the vertebrate including human possess a ubiquitous WASP-paralogue, N-WASP, which was originally described as neural-specific gene although expressed in nearly all cell types (Miki et al., 1996; Miki et al., 1998a). N-WASP commonly exists in an inactive confirmation in which Arp2/3 complex cannot interact with actin filament. Humans have four homologues of SCAR (HsSCAR1-4) (Bear et al., 1998) and one WAVE (Miki et al., 1998b) protein member representatives. Subcellular localization data of WASP family proteins underline the crucial role of these proteins in actin-based cell motility regulation (Papayannopoulos et al., 2005). The C-terminal VCA domain in all WASP family members is responsible for the activation of Arp2/3 complex to nucleate actin polymerization. On the other hand, the N-terminus contains variable domains that are considered to confer spatial and temporal regulation of Arp2/3 activating activity. The presence of Proline-rich regions, one or more WH2 (WASP homology-2) and WH1 (WASP homology-1) domain helps to interact with WIP (WASP interacting protein) and is required to stabilize WASP (Tyler et al., 2016). The GBD/CRIB domain bind to gtpase bound forms of Cdc42/Rac and alleviate auto inhibitory fold (Kim et al., 2000; Hemsath et al., 2005). The role of WASP family member as actin cytoskeleton regulators also links with the invasiveness and metastasis of cancer (review by (Kurisu and Takenawa, 2010)).

#### Activated Cdc42-Associated Kinase

ACK1, the only non-receptor Tyrosine kinase (or cytosolic Tyrosine kinase) is composed of diverse domains and its Tyrosine phosphorylation activates several effectors involved in cell proliferation and growth. ACK1 gets activated in response to multiple signals, majorly cell adhesion, growth factor receptors and hetero-trimeric G-protein coupled receptors (Prieto-Echague and Miller, 2011). The ACK1 possesses N-terminal catalytic kinase domain followed by a SH3 domain, C-terminal poly-Proline region (PPXY motif) and CRIB domain (Yang and Cerione, 1997). ACK1 was identified with some more domain regions which sets it apart from other non-receptor Tyrosine kinases (NRTKs) and includes at least eight distinct domains; the sterile α-motif (SAM), kinase or catalytic domain, SH3 domain, GTPase-binding domain, clathrin-interacting region, PPXY motif or WW domain-interacting region, an MIG6 homology region (MHR), also known as epidermal growth factor receptor (EGFR)-binding domain and an ubiquitin association (UBA) domain. It interacts only with Cdc42 and not Rac gtpase protein. Biochemical studies suggest that ACK1 strongly interacts with SH3 domain of Src family kinases (Src or Hck) via the C-terminal Proline-rich region (Yokoyama and Miller, 2003). Interaction of Cdc42 is required for auto phosphorylation whereas SH3 domain appears to function during auto inhibition (Galisteo et al., 2006). One of the substrates of ACK1 is WASP, which get phosphorylated and promotes its function (Yokoyama et al., 2005). The high expression of ACK1 during breast cancer makes it an appropriate marker for breast cancer detection. Apart from its role in breast cancer, ACK1 involved in stomach (Xu et al., 2015), hepatic (Xie et al., 2015; Wang et al., 2020), prostate (Furlow, 2006), ovarian, lung (Tan et al., 2014) and cervical cancer. The emergence of ACK1 as an oncogenic kinase has unraveled novel mechanisms by which dysregulated Tyrosine kinase signaling drives cancer progression through altered cellular homeostasis (Mahajan and Mahajan, 2015). Perhaps, recent data explains its function as an epigenetic regulator (Mahajan and Mahajan, 2015).

#### Myotonic Dystrophy-Related Cdc42-Binding Kinases Proteins

MRCK is primarily involved in actomyosin regulation by regulating Myosin II light chain (MLC) and has three isoforms (MRCKα, MRCKβ, MRCKγ) (Zhao et al., 1997; Leung et al., 1998; Ng et al., 2004) in human. MRCK, a CRIB containing effector is present in non-vertebrate and vertebrates but absent in lower eukaryotes and yeast (Pirone et al., 2001; Zhao and Manser, 2005). The Cdc42 regulated MRCK proteins are a subfamily of AGC (PKA, PAG and PKC) kinase family (Pearce et al., 2010). Apart from MRCK, Rho mediated signaling pathway for phosphorylation of MLC includes ROCK (RhoA binding coiled-coil containing kinases) (Olson, 2008) and CRIK (Citron rho interacting kinase) (201), which are functionally more established relative to MRCKs regulation mechanism. All three MRCKs have well conserved N-terminal kinase domain followed by a central linker linking to C-terminal with four domains. These four domains are protein kinase C conserved region (C1 domain) followed by PH domain, CH domain and lastly CRIB domain. C1 domain binds phorbol ester and might help in promoting kinase activation, while PH domain interacts mostly with lipid partners and leads to appropriate cellular localization of MRCKs (Loo et al., 2013). It has been expected that elevated MRCK expression might be prominent in invasiveness and metastasis of cancer, because actin myosin contractility is essential component of cell motility and is vital for cancer cell invasion and metastasis (Olson, 2008; Unbekandt and Olson, 2014; Zhao and Manser, 2015).

#### Mixed Lineage Kinases

MLKs are predominantly upstream kinases initiating the MAPK cascade, particularly the JNK (c-Jun N-terminal kinases). Four isoforms of MLKs (MLK1-4) are present in human and they are of immense interest because of their role in neurodegenerative conditions. The structural features of MLKs indicate N-terminal SH3 domain and kinase domain, a Leucine zipper, which connects the kinase domain to C-terminal CRIB/PBD domain (Dorow et al., 1993). Experimental evidences show that MLK3 interacts with GTP bound state of Cdc42 and Rac1 (Teramoto et al., 1996) and later on with RhoG as well (Zhang and Gallo, 2001; Wennerberg et al., 2002; Du et al., 2005). Earlier researches on gene silencing, genetically engineered mouse models and small molecule inhibitors suggest that MLKs are critical in tumor progression as well as in inflammatory processes (Handley et al., 2007; Gallo et al., 2020). Recent studies highlight the function of MLK3 in tumor cell proliferation, migration and invasion which opens the avenue for further research to investigate MLKs as potential therapeutic target for cancer treatment (Chadee, 2013; Gallo et al., 2020). Moreover, previous studies shows that the altered function/expression of MLK family of kinases leads to very wide spectrum of disorders (Handley et al., 2007; Craige et al., 2016).

#### Cdc42 Effector Protein

CEPs are also known as binder of Rho GTPases (Borg), with five isoforms present in humans. CEP/Borg protein family function as negative regulator of small GTPases. Borg1, Borg2, Borg4, and Borg5 (previously termed as MSE55) bind both TC10 GTPases and Cdc42, except Borg3, which only interacts with Cdc42 (Joberty et al., 1999). CEPs are structurally composed of a Cdc42 binding domain and two unique CI and CII domains (Hirsch et al., 2001), only exception is CEP5 that lacks CI domain. Alike PAK, ACK and WASP proteins, CEP family proteins also have the conserved consensus sequence at extended C-terminal CRIB domain (I-S-X-P-L-G-X-F-R-H-T-AA-H-AA-G-X-X-Gly-(X)_0–2_-D-AA-F-G-D-X-S-F-L, where AA represents an aliphatic amino acid) that can be involved in regulation of biological effects of CEP protein (Hirsch et al., 2001). Despite an earlier discovery, the molecular mechanism and functions CEPs or Borg family remain largely elusive. Interestingly, unlike other Cdc42 effectors, these genes are only present in vertebrates. However, recently researchers have investigated their role in tumor progression, regulation and function (Farrugia and Calvo, 2016; 2017).

#### Small Protein Effector of Cdc42

In humans, two members (SPEC1/Cdc42SE1 and SPEC2/Cdc42SE2) are found that contain a conserved N-terminal region and centrally located CRIB domain. Biochemical interaction studies show that it strongly interacts with Cdc42, weekly with Rac1 and not at all with RhoA (Pirone et al., 2000). One study reveals that three distinct regions (phosphoinositide-binding region within basic amino acids, N-terminal to CRIB sequence) within SPECs are likely to be involved in early contractile events in phagocytosis (Ching et al., 2007). SPECs have been shown to play an important role in Cdc42-mediated F-actin accumulation at immunological synapse (Ching et al., 2005). In disease connection, SPEC1 was down regulated during skin cancer to promote tumorigenesis, and thus proposed to be as an important marker of skin cancer progression (Kalailingam et al., 2019). This family of proteins is yet to be investigated for regulation, expression and function in details to chronology connect.

#### Gene33

Gene 33, also called as mitogen-inducible gene-6 (Mig-6) or ERFI1 is an immediate early gene that is induced transcriptionally by several extracellular stimuli. Physiological function of Gene-33 remains unclear but commonly occurring chronic stress stimuli (mechanical strain, vasoactive peptides and diabetic nephropathy) increase its mRNA levels in the cells (Makkinje et al., 2000; Park et al., 2017). The structure of Gene33 resembles an adaptor protein capable of binding monomeric gtpase (HsCdc42) *in vivo* and *in vitro*. Gene33/Mig-6 is a negative regulator of EGF signaling (Park et al., 2015), and Mig-6 inhibits Cdc42 signaling which is critical for Mig-6 function to suppress cell migration. The dysregulation of Cdc42 mediated Gene33 pathway may play a critical role in cancer development (Jiang et al., 2016).

#### PAR-6

Structurally PAR-6 have pseudo-CRIB domain in comparison to other CRIB containing effectors. PAR-6 functions mainly in determining cell polarity and are conserved throughout metazoans where it is a substrate of aPKC (atypical protein kinase C) (Izumi et al., 1998; Lin et al., 2000). The Cdc42-PAR-6-aPKC complex involved with PAR-3 localizes in epithelial cells’ apical rings and E-cadherin at adherens junction (Yamanaka et al., 2001; Atwood et al., 2007; Thompson, 2021). PAR-6 has three isoforms in humans that are involved in cell polarity regulated by Cdc42 (Joberty et al., 2000; Macara, 2004).

## Discussion

Understanding the domain architecture serves as a crucial link to decipher a protein’s molecular mechanism in intracellular signaling cascades. Several protein domains and their relation to signaling components present in humans are entirely missing from the best-studied model organism or pathogens and vice versa. The signaling pathways involving Cdc42 and Rac GTPases are conserved in all eukaryotes but their interactive proteins (CRIB) are not annotated or characterized in lower eukaryotes like protozoan parasites. The current study identifies and describes the CRIB domain containing protein families and their possible role (according to domain architechture) as effector proteins for cytoskeletal regulation. The structural similarities, combination of regulatory domains, and their putative/observed functions in lower eukaryotic pathogens have been accounted here. Genome and proteome information allows us for a better understanding of pathogenic processes and consequently help improve the prevention, diagnosis, and treatment of the diseases.

The evolutionary divergence shows that owing to their ancient origin, PAK and WASP families are termed as conventional effector molecules. They contain a conserved CRIB motif with its extended region known as PBD (in PAK) and GBD (in WASP), crucial for cytoskeleton dynamics. However, coronin protein family is an exception that binds GDP-Rac/Cdc42 in *Dictyostelium* (Swaminathan et al., 2014). However, earlier studies on CRIB effector proteins; coronin have not been included in the list of Cdc42 effectors, despite their well-defined CRIB motif shown in the structural and functional studies (Swaminathan et al., 2014). The coronin family proteins must be include in the CRIB effectors now. The CRIB proteins in protozoan parasites is not investigated in any capacity and reported here which opens an avenue for their functional and biophysical studies as potential drug candidates against host.

The evolution of the other reported effector protein families started later on as seen in worm, flies, frog, and human, displaying a direct correlation between the increase in the complexity of the organism with the increase in several members of each family. These effector families: ACK, MRCK, MLK, SPEC, CEP, PAR-6 now classified as non-conventional Cdc42/Rac effectors. The highest evolved organism, human, consists of conventional and non-conventional Cdc42/Rac effector protein members along with some other proteins like IQGAPs (IQGAP1-3), IRSp53 etc ((Pichaud et al., 2019) Supplementary Information).

Structural insights into the CRIB effector protein emphasizes that the C-terminal inhibitory switch (IS) domain responsible for sustaining it in an auto-inhibited basal state, is well-conserved during evolution (Hoffman and Cerione, 2000; Kim et al., 2000; Lei et al., 2000). Depending on the context of low or high-affinity binding, GBD/PBD domain adopts related but distinct folds (Rudolph et al., 1998; Kim et al., 2000). When the GBD domain is in the free state, it looks largely unstructured, while in an auto-inhibited state, it forms an β-hairpin structure following the conserved CRIB motif and a central three-helix bundle. However, during the interaction, the PBD/GBD forms high-affinity complex with their respective G-proteins, in which the unstructured region becomes structured (Hoffman and Cerione, 2000; Kim et al., 2000; Lei et al., 2000). Similarly, the Cdc42-CRIB motif interaction occurs between the β2 strand of Cdc42 through the formation of an intermolecular β-sheet (Abdul-Manan et al., 1999; Mott et al., 1999; Hoffman and Cerione, 2000; Morreale et al., 2000). The GBD domains (except CRIB motif) display divergence in their C-terminal regions with several binding mode variations, possibly determining the specificity of interaction with effector protein. Association of GBD/PBD domains with Cdc42/Rac, instigates a stagy change in the conformation that refolds the IS domain while unfolding the rest of the structure (Hoffman and Cerione, 2000). The CRIB motif interacts with switch I and II regions of GTPases (Stevens et al., 1999). It has been observed generally that two histidine at the C-terminal and one at sixth positions are well conserved, and along with the adjacent α-helix mediate sensitivity to the nucleotide switch. This establishes that the CRIB motif prefers GTP-loaded GTPases and exhibits decreased binding activity with GDP form. Apart from GTP bound state, the flanking sequence of the CRIB motif also determines Cdc42/Rac binding specificity (Pirone et al., 2001; Owen and Mott, 2018). The typical example of such specificity is an autoinhibitory domain of PAKs located C-terminally to the CRIB/PBD domain (Lei et al., 2000; Kim et al., 2016). The new concepts of the intrinsically disordered region (IDR) have also emerged, which highlight that apart from modular and defined domain, the basic rich (BR) regions like poly-Proline leads to the structure-function paradigm in CRIB containing effector proteins (Papayannopoulos et al., 2005; Pang and Zhou, 2016; Owen and Mott, 2018).

The structural component of CRIB containing effector proteins also highlights that apart from CRIB domain and signature domain of that family, various accessory domains like, Proline-rich sequences, PH domain, AID domain, and others are crucial for regulation, activation, subcellular localization and specificity of substrate to perform definite functions. The conventional or non-conventional protein family’s structures share numerous parallelisms in signaling and arrangement in course of evolution. The notable fact that lower eukaryotes represent functional equivalents/counterparts with high sequence divergence in intrinsically regions of proteins. The putative proteins identified in this study from gastric protozoan pathogens and kinetoplastids will be of great importance to study experimentally and some can be used as therapeutic targets against neglected tropical diseases cause by them in their human host. The putative proteins of occasional parasite social amoeba can also be utilized to study the function and regulation of activation of CRIB domain containing effector proteins to understand the role of comparative to the human homologues. Two unique domain combination protein of *Acanthamoeba* are of great importance in light of drug targets.

Taken together, we would like to summarize that CRIB motif is ancient in origin and it has conventional effector family protein members present in model organism and protozoan parasites. The primary classification and identified protein awaits experimental confirmation for being potential drug targets and key players for the survival of the pathogen.

## Methods

**FIGURE 6:**
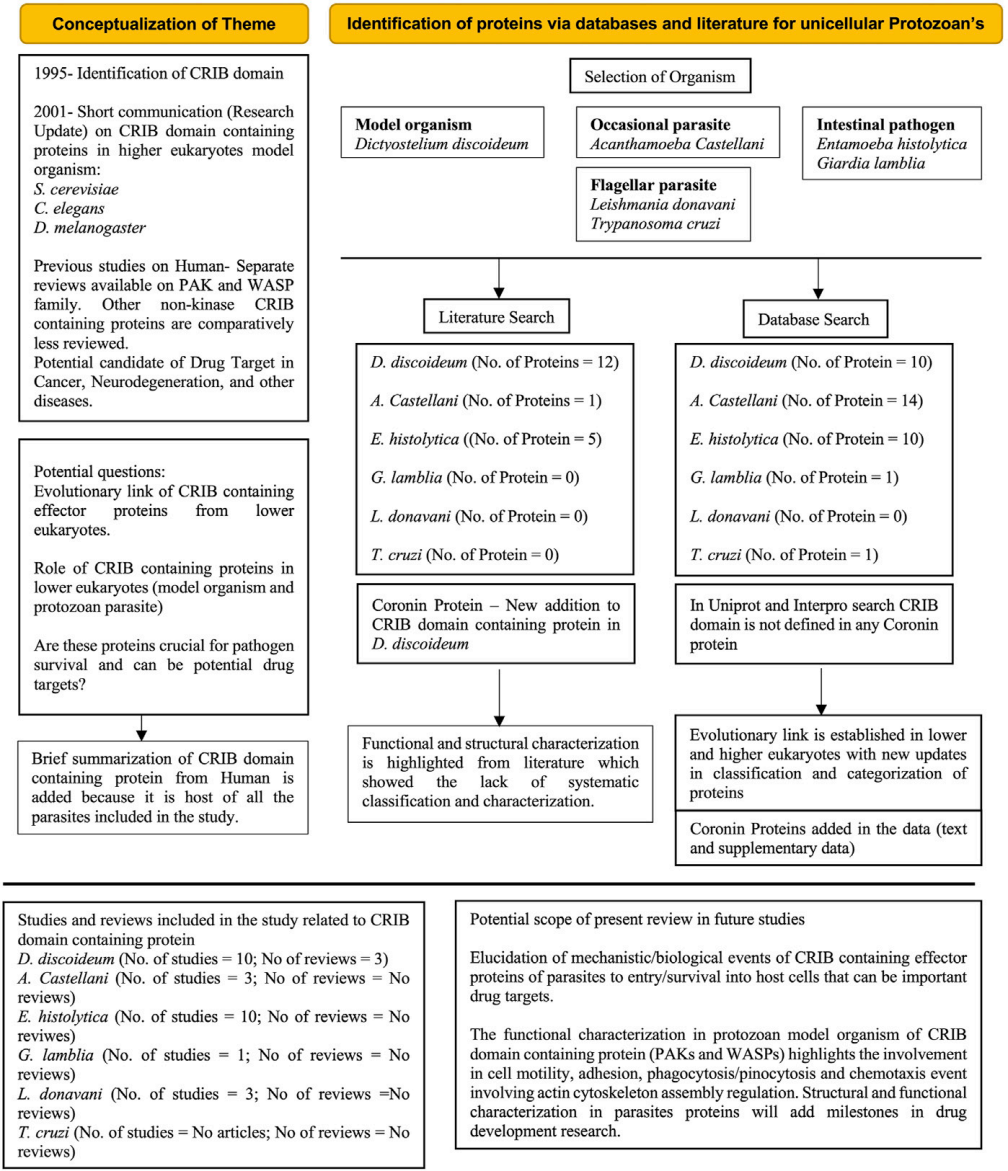
PRISMA flow diagram (Reference) explains the conceptualization, brief overview of database and literature search and outcomes of systematic review. The numbers of studies and reviews mentioned in box purely accounting CRIB containing proteins about particular organism. The future scope of systematic review explained in key bullets. Coronin proteins which are unique and proposed to be included in this review based on individual protein characterization (Protein ID’s are included in Supplementary Information).

We first tabulated the various types of CRIB-domain containing proteins from the available literature. We used the InterPro database (Blum et al., 2021) to carry out a domain-based search for all the types of CRIB domains against the selected organisms. Our data consists of proteins searched across the proteome of the following organisms: *Dictyostelium discoideum*, *Acanthamoeba castellani*, *Entamoeba histolytica*, *Trypanosoma cruzi*, *Leishmania donovani*, and *Giardia lamblia*. We also used the proteins from humans for comparative purposes using their domain architecture (Figure 6) (Page et al., 2021). The protein ID’s used in the manuscript was procured from the UniProt database for universal usage (UniProt, 2021). Once the proteins from all the organisms were fished out, we used Phyre2 software to determine their structure homology to better classify them (Kelley et al., 2015). We performed a manual sequence analysis as well to corroborate the presence of variant CRIB domains, otherwise missed out by the software, as well. Sequence alignment was performed using the ClustalW software and the final Figure was prepared with the Sequence Manipulation Suite2 website (Thompson et al., 1994; Stothard, 2000).

## Data Availability

The original contributions presented in the study are included in the article/Supplementary Material, further inquiries can be directed to the corresponding author.
