# Supplementary material for: Cdc42/Rac Interactive Binding Containing Effector Proteins in Unicellular Protozoans With Reference to Human Host: Locks of the Rho Signaling
Source: Front Genet. 2022 Feb 2;13:781885. doi: 10.3389/fgene.2022.781885 (PMC8847673; doi:10.3389/fgene.2022.781885)
Supplement: Supplementary file 1 [file DataSheet1.docx]

**Supplementary Material**

**Table 1:** List of the CRIB domain containing protein in model organism (*Dictyostellium discoideum*) and protozoan parasites (*Acanthamoeba castellani, Entamoeba histolytica, Giardia lambia, Trypanosoma cruzi,* and *Leishmania donavani*) with characterized and putative names with key references of structure and functional role of protein.

| SN | Protein family | Uniprot ID | Protein Characterization | Reference |
| --- | --- | --- | --- | --- |
| *Dictyostelium discoideum* | | | | |
|  | PAK | | | |
| 1 |  | Q55D99 | PAKa | 47 |
| 2 |  | Q869N2 | PAKb/MIHCK. Two isoforms (Q869N2.2- 413-440 aa missing) | 42, 45 |
| 3 |  | Q55GV3 | PAKc | 41 |
| 4 |  | Q55DD4 | PAKd | 40, 43 |
| 5 |  | Q869T7 | PAKf | Uncharacterized, This paper |
| 6 |  | Q54RV3 | PAKg | Uncharacterized, This paper |
|  | Ser/thr Kinase | | | |
|  |  | Q54B33 | ser/thr protein kinase (PAKe) | Pseudo PAK like EhPAK1, Uncharacterized, This Paper |
|  |  | Q556S2 | ser/thr protein kinase (PAKh) | Pseudo PAK like EhPAK1, Uncharacterized, This paper |
|  | WASP |  | |  |
| 1 |  | Q9GSG9 | WASPA (wasA gene) | 48 |
|  | WASP related | | | |
| 1 |  | Q7KWP7 | WASP like protein B (wasB gene) | 46 |
| 2 |  | Q54QH4 | WASP like protein C (wasC gene) | Uncharacterized, This paper |
|  | Gelsolin Like Protein | | | |
| 1 |  | Q551I6 | Gelsolin like protein/Non-specific ser/thr kinase | Uncharacterized, This paper |
|  | Coronin |  | |  |
| 1 |  | P27133 | CoroninA (corA gene) | 6, 7, 44 |
| 2 |  | Q55E54 | CoroninB (corB gene) | 6, 38, 39 |
| 3 |  |  | Villidin | 8, 11 |

| *Acanthamoeba castellani* | | | | |
| --- | --- | --- | --- | --- |
|  | PAK | | | |
| 1 |  | **Q93107** | MIHCK/PAK | 50, 51 |
|  | PAK related protein | | | |
| 1 |  | L8GPX2 | PAK like protein 1 | MIHCK/Pak like, Uncharacterized, This paper |
| 2 |  | L8H707 | PAK like protein 2 | Putative PAK, Uncharacterized, This paper |
| 3 |  | L8GW48 | PAK like protein 3 | PAKa subfamily protein kinase, Uncharacterized, This paper |
|  | PAK like ser/thr kinase protein | | | |
| 1 |  | L8HHB6 | Non-specific ser/thr protein kinase | Uncharacterized, This paper |
| 2 |  | L8GCL9 | Non-specific ser/thr protein kinase | Uncharacterized, This paper |
| 3 |  | L8GVV0 | Ser/threonine kinase | Uncharacterized, This paper |
| 4 |  | L8HET0 | Ser/threonine kinase | Uncharacterized, This paper |
|  | WASP related protein | | | |
| 1 |  | L8H2P8 | WASP like protein 1 | WH1 domain containing protein, Uncharacterized, This paper |
| 2 |  | L8H6L6 | WASP like protein 2 | WH1 domain containing protein, Uncharacterized, This paper |
| 3 |  | L8H3X7 | WASP like protein 3 | WH1 domain containing protein, Uncharacterized, This paper |
| 4 |  | L8GYC7 | WASP like protein 4 | WH2 domain containing protein, Uncharacterized, This paper |
|  | Others | | | |
| 1 |  | L8H695 | CRIB domain/leucine rich repeat containing protein | Uncharacterized, This paper |
| 2 |  | L8GKX1 | CRIB domain/tudor domain containing protein | Uncharacterized, This paper |
|  | Coronin | | | |
| 1 |  | L8GFZ9 | Coronin | Uncharacterized, 12 |
| 2 |  |  |  |  |

| *Entamoeba histolytica* | | | | |
| --- | --- | --- | --- | --- |
|  | PAK | | | |
| 1 |  | Q24848 | Pseudo PAK/PAK1 | No CRIB, 32, 33 |
| 2 |  | C4LT57 | PAK2 | 31, 34 |
| 3 |  | Q5FZP2 | PAK3 | 34, 36 |
| 4 |  | C4LSU1 | PAK4 | 34, 35 |
| 5 |  | C4MBQ2 | PAK5 | 34, 35 |
| 6 |  | C4LVP2 | PAK6 | Uncharacterized, 34, This paper |
| 7 |  | C4M1H4 | PAK7 | Uncharacterized, 34, This paper |
|  | Others | | | |
| 1 |  | C4M2L6 | C-ter WASP like CRIB domain containing protein | Uncharacterized, This paper |
| 2 |  | C4LZJ6 | C-ter WASP like CRIB domain containing protein | Uncharacterized, This paper |
| 3 |  | C4M0R3 | C-ter WASP like CRIB domain containing protein | Uncharacterized, This paper |
|  | Coronin | | | |
| 1 |  | C4M137 | Coronin (CRN12b) | Uncharacterized, 6, 12, 13 This paper |
| 2 |  | C4M943 | Coronin (CRN12a) | Uncharacterized, 6, 12, 13 This paper |
| 3 |  | C4M5U0 | Coronin7 (Class 3) | Uncharacterized, 6, 12, 13, This paper |
| 4 |  | C4M5M6 | Coronin (Class 4) | Uncharacterized, 6, 12, This paper |

| *Giardia lambia* | | | | |
| --- | --- | --- | --- | --- |
| 1 |  | C6LUS0 | Non-specific serine/threonine protein kinase | Uncharacterized, This paper |
| *Trypanosoma cruzi* | | | | |
| 1 |  | Q4DHX7 | CRIB domain containing protein | Uncharacterized, This paper |
|  | Coronin | | | |
| 1 |  | Q4DEX0 | Coronin | Uncharacterized, 6, This paper |
| 2 |  | Q4D4X6 | Coronin | Uncharacterized, 6, 12, 18 This paper |
| *Leishmania donavani* | | | | |
| 1 |  | Q4QEZ0 | PAK like kinase domain containing protein | No CRIB, Uncharacterized, This paper |
|  | Coronin | | | |
| 1 |  | Q3T1U8 | Coronin (CRN12, Class 3) | 13, 6, 12,18 |

**Table 2:** List of the CRIB domain containing protein in human host with characterized and putative names with key references of structure and functional role of protein.

| SN |  | Uniprot ID | Protein Characterization | Key Reference |
| --- | --- | --- | --- | --- |
| *Homo Sapiens* | | | | |
|  | PAK | | | |
| 1 |  | B3KNX7  Q13153 | PAK1 | 59, 65, 66 |
| 2 |  | Q13177 | PAK2 | 69, 59, 65 |
| 3 |  | O75914  A0A087X294 | PAK3 | 64, 59, 65 |
| 4 |  | O96013 | PAK4 | 60, 59, 65 |
| 5 |  | Q9P286 | PAK5 | 61, 59, 62, 65 |
| 6 |  | Q9NQU5 | PAK6 | 59, 65 |
|  | MRCK | | | |
| 1 |  | Q5VT25  A0A0A0MRJ0  A0A0A0MRJ1 | MRCKα | 22, 24, 27 |
| 2 |  | Q9Y5S2 | MRCKβ | 23, 24, 27 |
| 3 |  | Q6DT37 | MRCKγ | 21, 24 |
|  | MLK | | | |
| 1 |  | P80192  J3KPI6  G3V347  A0A087WW79  G3V4P9 | MLK1/MAP3K9 | 54, 55 |
| 2 |  | Q02779 | MLK2/MAP3K10 | 52, 53, 54 |
| 3 |  | Q16584 | MLK3 | 28 |
| 4 |  | Q5TCX8 | MLK4 | 30 |
|  | ACK | | | |
|  |  | Q07912 | ACK1 | 25, 26, 63 |
|  | WASP | | | |
| 1 |  | P42768 | WASP | 56, 57, 58 |
| 2 |  | O00401 | N-WASP | 68 |
|  | Coronin | | | |
| 1 |  | [P31146](https://www.uniprot.org/uniprot/P31146) | Coronin1A | 2, 1, 5, 6 |
| 2 |  | [Q9BR76](https://www.uniprot.org/uniprot/Q9BR76) | Coronin1B | 14, 3, 1, 5, 6 |
| 3 |  | [Q9ULV4](https://www.uniprot.org/uniprot/Q9ULV4) | Coronin1C | 4, 1, 5, 6,9 |
| 4 |  | Q6QEF8 | Coronin1D/Coronin6 | 5, 6 |
|  |  | Q92828 | Coronin2A | 15, 16,12 |
| 5 |  | [Q9UQ03](https://www.uniprot.org/uniprot/Q9UQ03) | Coronin2B | 16,1, 5, 6 |
| 6 |  | [P57737](https://www.uniprot.org/uniprot/P57737) | Coronin7 | 10, 1, 5, 6, 12 |
|  | CEP | | | |
| 1 |  | Q00587 | CEP1/Borg5 | 19, 20 |
| 2 |  | O14613 | CEP2/Borg1 | 19, 20 |
| 3 |  | Q9UKI2  C9JEZ4 | CEP3/Borg2 | 19, 20 |
| 4 |  | Q9H3Q1 | CEP4/Borg4 | 19, 20 |
| 5 |  | Q6NZY7 | CEP5/Borg3 | 19, 20 |
|  | SPEC | | | |
| 1 |  | Q9NRR8 | SPEC1 | 29 |
| 2 |  | Q9NRR3  D6REL0 | SPEC2 | 29 |
|  | Gene33 | | | |
| 1 |  | Q9UJM3  I6S2Y9 B3KTV8 |  |  |
|  | PAR6 (pseudo-CRIB) | | | |
| 1 |  | Q9NPB6 | PAR6α | 67, 70, 71 |
| 2 |  | Q9BYG5 | PAR6β | 73, 71, 74 |
| 3 |  | Q9BYG4 | PAR6γ | 72, 73, 71 |
|  | Others | | | |
|  |  | IQGAPS |  | 75 |
|  |  | IRSp53 |  | 75 |

**References**

1. Uetrecht AC, Bear JE. Coronins: the return of the crown. Trends Cell Biol. 2006 Aug;16(8):421-6. doi: 10.1016/j.tcb.2006.06.002. Epub 2006 Jun 27. PMID: 16806932.
2. Suzuki K, Nishihata J, Arai Y, Honma N, Yamamoto K, Irimura T, Toyoshima S. Molecular cloning of a novel actin-binding protein, p57, with a WD repeat and a leucine zipper motif. FEBS Lett. 1995 May 15;364(3):283-8. doi: 10.1016/0014-5793(95)00393-n. PMID: 7758584.
3. Ojeda V, Castro-Castro A, Bustelo XR. Coronin1 proteins dictate rac1 intracellular dynamics and cytoskeletal output. Mol Cell Biol. 2014 Sep 15;34(18):3388-406. doi: 10.1128/MCB.00347-14. Epub 2014 Jun 30. PMID: 24980436; PMCID: PMC4135624.
4. Iizaka M, Han HJ, Akashi H, Furukawa Y, Nakajima Y, Sugano S, Ogawa M, Nakamura Y. Isolation and chromosomal assignment of a novel human gene, CORO1C, homologous to coronin-like actin-binding proteins. Cytogenet Cell Genet. 2000;88(3-4):221-4. doi: 10.1159/000015555. PMID: 10828594.
5. Xavier CP, Eichinger L, Fernandez MP, Morgan RO, Clemen CS. Evolutionary and functional diversity of coronin proteins. Subcell Biochem. 2008;48:98-109. doi: 10.1007/978-0-387-09595-0_9. PMID: 18925374.
6. Morgan RO, Fernandez MP. Molecular phylogeny and evolution of the coronin gene family. Subcell Biochem. 2008;48:41-55. doi: 10.1007/978-0-387-09595-0_5. PMID: 18925370.
7. de Hostos EL, Bradtke B, Lottspeich F, Guggenheim R, Gerisch G: Coronin, an actin binding protein of Dictyostelium discoideum localized to cell surface projections, has sequence similarities to G protein beta subunits. EMBO J. 1991, 10: 4097-4104.
8. Gloss A, Rivero F, Khaire N, Muller R, Loomis WF, Schleicher M, Noegel AA: Villidin, a novel WD-repeat and villin-related protein from Dictyostelium, is associated with membranes and the cytoskeleton. Mol Biol Cell. 2003, 14: 2716-2727. 10.1091/mbc.E02-12-0827.
9. Xavier CP, Rastetter RH, Stumpf M, Rosentreter A, Muller R, Reimann J, Cornfine S, Linder S, van Vliet V, Hofmann A, Morgan RO, Fernandez MP, Schroder R, Noegel AA, Clemen CS: Structural and functional diversity of novel coronin 1C (CRN2) isoforms in muscle. J Mol Biol. 2009, 393: 287-299. 10.1016/j.jmb.2009.07.079.
10. Rybakin V, Stumpf M, Schulze A, Majoul IV, Noegel AA, Hasse A. Coronin 7, the mammalian POD-1 homologue, localizes to the Golgi apparatus. FEBS Lett. 2004 Aug 27;573(1-3):161-7. doi: 10.1016/j.febslet.2004.07.066. PMID: 15327992.
11. Archer SK, Claudianos C, Campbell HD: Evolution of the gelsolin family of actin-binding proteins as novel transcriptional coactivators. Bioessays. 2005, 27: 388-396. 10.1002/bies.20200.
12. Eckert, C., Hammesfahr, B. & Kollmar, M. A holistic phylogeny of the coronin gene family reveals an ancient origin of the tandem-coronin, defines a new subfamily, and predicts protein function. BMC Evol Biol 11, 268 (2011). https://doi.org/10.1186/1471-2148-11-268
13. Nayak RC, Sahasrabuddhe AA, Bajpai VK, Gupta CM. A novel homologue of coronin colocalizes with actin in filament-like structures in Leishmania. Mol Biochem Parasitol. 2005 Oct;143(2):152-64. doi: 10.1016/j.molbiopara.2005.06.001. PMID: 16024104.
14. Parente JA Jr, Chen X, Zhou C, Petropoulos AC, Chew CS. 1999. Isolation, cloning, and characterization of a new mammalian coronin family member, coroninse, which is regulated within the protein kinase C signaling pathway. J Biol Chem 274:3017 – 3025.
15. Zaphiropoulos PG, Toftgard R. 1996. cDNA cloning of a novel WD repeat protein mapping to the 9q22.3 chromosomal region. DNA Cell Biol 15:1049 – 1056.
16. Nakamura T, Takeuchi K, Muraoka S, Takezoe H, Takahashi N, et al. 1999. A neurally enriched coronin-like protein, ClipinC, is a novel candidate for an actin cytoskeleton-cortical membrane-linking protein. J Biol Chem 274:13322 – 13327.
17. Rath PP, Gourinath S. The actin cytoskeleton orchestra in Entamoeba histolytica. Proteins. 2020 Oct;88(10):1361-1375. doi: 10.1002/prot.25955. Epub 2020 Jun 25. PMID: 32506560.
18. Gupta CM, Ambaru B and Bajaj R (2020) Emerging Functions of Actins and Actin Binding Proteins in Trypanosomatids. Front. Cell Dev. Biol. 8:587685. doi: 10.3389/fcell.2020.587685
19. Joberty, G., Perlungher, R.R. and Macara, I.G. (1999) The Borgs, a new family of Cdc42 and TC10 GTPase-interacting proteins. Mol. Cell. Biol. 19, 6585–6597 doi:10.1128/MCB.19.10.6585
20. Farrugia AJ, Calvo F. The Borg family of Cdc42 effector proteins Cdc42EP1-5. Biochem Soc Trans. 2016 Dec 15;44(6):1709-1716. doi: 10.1042/BST20160219. PMID: 27913681; PMCID: PMC5134998.
21. Ng Y, Tan I, Lim L, Leung T (2004) Expression of the human myotonic dystrophy kinase-related Cdc42-binding kinase gamma is regulated by promoter DNA methylation and Sp1 binding. J Biol Chem 279:34156–34164
22. Tan I, Cheong A, Lim L, Leung T (2003) Genomic organization of human myotonic dystrophy kinase-related Cdc42-binding kinase α reveals multiple alternative splicing and functional diversity. Gene 304:107–115
23. Moncrieff CL, Bailey ME, Morrison N, Johnson KJ (1999) Cloning and chromosomal localization of human Cdc42-binding protein kinase beta. Genomics 57:297–300
24. Unbekandt M, Olson MF. The actin-myosin regulatory MRCK kinases: regulation, biological functions and associations with human cancer. J Mol Med (Berl). 2014 Mar;92(3):217-25. doi: 10.1007/s00109-014-1133-6. Epub 2014 Feb 20. PMID: 24553779; PMCID: PMC3940853.
25. Manser E, Leung T, Salihuddin H, Tan L, Lim L. A non-receptor tyrosine kinase that inhibits the GTPase activity of p21cdc42. Nature. 1993 May 27;363(6427):364-7. doi: 10.1038/363364a0. PMID: 8497321.
26. Fox M, Crafter C, Owen D. The non-receptor tyrosine kinase ACK: regulatory mechanisms, signalling pathways and opportunities for attACKing cancer. Biochem Soc Trans. 2019 Dec 20;47(6):1715-1731. doi: 10.1042/BST20190176. PMID: 31845724.
27. Leung T, Chen XQ, Tan I, Manser E, Lim L. Myotonic dystrophy kinase-related Cdc42-binding kinase acts as a Cdc42 effector in promoting cytoskeletal reorganization. Mol Cell Biol. 1998 Jan;18(1):130-40. doi: 10.1128/MCB.18.1.130. PMID: 9418861; PMCID: PMC121465.
28. Teramoto H, Coso OA, Miyata H, Igishi T, Miki T, Gutkind JS. Signaling from the small GTP-binding proteins Rac1 and Cdc42 to the c-Jun N-terminal kinase/stress-activated protein kinase pathway. A role for mixed lineage kinase 3/protein-tyrosine kinase 1, a novel member of the mixed lineage kinase family. J Biol Chem. 1996 Nov 1;271(44):27225-8. doi: 10.1074/jbc.271.44.27225. PMID: 8910292.
29. Pirone DM, Fukuhara S, Gutkind JS, Burbelo PD. SPECs, small binding proteins for Cdc42. J Biol Chem. 2000 Jul 28;275(30):22650-6. doi: 10.1074/jbc.M002832200. PMID: 10816584.
30. Kashuba VI, Grigorieva EV, Kvasha SM, Pavlova TV, Grigoriev V, Protopopov A, Kharchenko O, Gizatullin R, Rynditch AV, Zabarovsky ER. Cloning and Initial Functional Characterization of Mlk4α and Mlk4β. Genomics Insights. 2011 Mar 22;4:1-12. doi: 10.4137/GEI.S6092. PMID: 26217104; PMCID: PMC4510602.
31. Arias-Romero LE, de Jesús Almáraz-Barrera M, Díaz-Valencia JD, Rojo-Domínguez A, Hernandez-Rivas R, Vargas M. EhPAK2, a novel p21-activated kinase, is required for collagen invasion and capping in Entamoeba histolytica. Mol Biochem Parasitol. 2006 Sep;149(1):17-26. doi: 10.1016/j.molbiopara.2006.04.001. Epub 2006 May 2. PMID: 16716419.
32. Labruyère E, Zimmer C, Galy V, Olivo-Marin JC, Guillén N. EhPAK, a member of the p21-activated kinase family, is involved in the control of Entamoeba histolytica migration and phagocytosis. J Cell Sci. 2003 Jan 1;116(Pt 1):61-71. doi: 10.1242/jcs.00190. PMID: 12456716.
33. Labruyere E, Galy V, Sansonetti P, Guillén N. Distribution of a potential p21-activated serine/threonine kinase (PAK) in Entamoeba histolytica. Arch Med Res. 2000 Jul-Aug;31(4 Suppl):S128-30. doi: 10.1016/s0188-4409(00)00143-0. PMID: 11070253.
34. Anamika K, Bhattacharya A, Srinivasan N. Analysis of the protein kinome of Entamoeba histolytica. Proteins. 2008 May 1;71(2):995-1006. doi: 10.1002/prot.21790. PMID: 18004777.
35. Bosch DE, Siderovski DP. Entamoeba histolytica RacC selectively engages p21-activated kinase effectors. Biochemistry. 2015 Jan 20;54(2):404-12. doi: 10.1021/bi501226f. Epub 2015 Jan 2. PMID: 25529118; PMCID: PMC4303316.
36. Dutta S, Sardar A, Ray D, Raha S. Molecular and functional characterization of EhPAK3, a p21 activated kinase from Entamoeba histolytica. Gene. 2007 Nov 1;402(1-2):57-67. doi: 10.1016/j.gene.2007.07.022. Epub 2007 Aug 2. PMID: 17761392.
37. Gangopadhyay SS, Ray SS, Sinha P, Lohia A. Unusual genome organisation in Entamoeba histolytica leads to two overlapping transcripts. Mol Biochem Parasitol. 1997 Oct;89(1):73-83. doi: 10.1016/s0166-6851(97)00110-2. PMID: 9297702.
38. Swaminathan K, Stumpf M, Müller R, Horn AC, Schmidbauer J, Eichinger L, Müller-Taubenberger A, Faix J, Noegel AA. Coronin7 regulates WASP and SCAR through CRIB mediated interaction with Rac proteins. Sci Rep. 2015 Sep 28;5:14437. doi: 10.1038/srep14437. PMID: 26411260; PMCID: PMC4585930.
39. Shina MC, Unal C, Eichinger L, Müller-Taubenberger A, Schleicher M, Steinert M, Noegel AA. A Coronin7 homolog with functions in actin-driven processes. J Biol Chem. 2010 Mar 19;285(12):9249-61. doi: 10.1074/jbc.M109.083725. Epub 2010 Jan 12. PMID: 20071332; PMCID: PMC2838343.
40. Phillips JE, Gomer RH. The p21-activated kinase (PAK) family member PakD is required for chemorepulsion and proliferation inhibition by autocrine signals in Dictyostelium discoideum. PLoS One. 2014 May 5;9(5):e96633. doi: 10.1371/journal.pone.0096633. PMID: 24797076; PMCID: PMC4010531.
41. Lee S, Rivero F, Park KC, Huang E, Funamoto S, Firtel RA. Dictyostelium PAKc is required for proper chemotaxis. Mol Biol Cell. 2004 Dec;15(12):5456-69. doi: 10.1091/mbc.e04-04-0323. Epub 2004 Oct 13. PMID: 15483055; PMCID: PMC532025.
42. Lee SF, Egelhoff TT, Mahasneh A, Côté GP. Cloning and characterization of a Dictyostelium myosin I heavy chain kinase activated by Cdc42 and Rac. J Biol Chem. 1996 Oct 25;271(43):27044-8. doi: 10.1074/jbc.271.43.27044. PMID: 8900194.
43. Garcia M, Ray S, Brown I, Irom J, Brazill D. PakD, a putative p21-activated protein kinase in Dictyostelium discoideum, regulates actin. Eukaryot Cell. 2014 Jan;13(1):119-26. doi: 10.1128/EC.00216-13. Epub 2013 Nov 15. PMID: 24243792; PMCID: PMC3910960.
44. Swaminathan K, Müller-Taubenberger A, Faix J, Rivero F, Noegel AA. A Cdc42- and Rac-interactive binding (CRIB) domain mediates functions of coronin. Proc Natl Acad Sci U S A. 2014 Jan 7;111(1):E25-33. doi: 10.1073/pnas.1315368111. Epub 2013 Dec 17. PMID: 24347642; PMCID: PMC3890859.
45. de la Roche M, Mahasneh A, Lee SF, Rivero F, Côté GP. Cellular distribution and functions of wild-type and constitutively activated Dictyostelium PakB. Mol Biol Cell. 2005 Jan;16(1):238-47. doi: 10.1091/mbc.e04-06-0534. Epub 2004 Oct 27. PMID: 15509655; PMCID: PMC539168.
46. Chung CY, Feoktistov A, Hollingsworth RJ, Rivero F, Mandel NS. An attenuating role of a WASP-related protein, WASP-B, in the regulation of F-actin polymerization and pseudopod formation via the regulation of RacC during Dictyostelium chemotaxis. Biochem Biophys Res Commun. 2013 Jul 12;436(4):719-24. doi: 10.1016/j.bbrc.2013.06.022. Epub 2013 Jun 17. PMID: 23791739; PMCID: PMC3799868.
47. Chung CY, Firtel RA. PAKa, a putative PAK family member, is required for cytokinesis and the regulation of the cytoskeleton in Dictyostelium discoideum cells during chemotaxis. J Cell Biol. 1999 Nov 1;147(3):559-76. doi: 10.1083/jcb.147.3.559. PMID: 10545500; PMCID: PMC2151188.
48. Myers SA, Han JW, Lee Y, Firtel RA, Chung CY. A Dictyostelium homologue of WASP is required for polarized F-actin assembly during chemotaxis. Mol Biol Cell. 2005 May;16(5):2191-206. doi: 10.1091/mbc.e04-09-0844. Epub 2005 Feb 23. PMID: 15728724; PMCID: PMC1087228.
49. Müller-Taubenberger A, Bretschneider T, Faix J, Konzok A, Simmeth E, Weber I. Differential localization of the Dictyostelium kinase DPAKa during cytokinesis and cell migration. J Muscle Res Cell Motil. 2002;23(7-8):751-63. doi: 10.1023/a:1024475628061. PMID: 12952073.
50. Brzeska H, Young R, Knaus U, Korn ED. Myosin I heavy chain kinase: cloning of the full-length gene and acidic lipid-dependent activation by Rac and Cdc42. Proc Natl Acad Sci U S A. 1999 Jan 19;96(2):394-9. doi: 10.1073/pnas.96.2.394. PMID: 9892644; PMCID: PMC15147.
51. Brzeska H, Young R, Tan C, Szczepanowska J, Korn ED. Calmodulin-binding and autoinhibitory domains of Acanthamoeba myosin I heavy chain kinase, a p21-activated kinase (PAK). J Biol Chem. 2001 Dec 14;276(50):47468-73. doi: 10.1074/jbc.M108957200. Epub 2001 Sep 28. PMID: 11579107.
52. Katoh M, Hirai M, Sugimura T, Terada M. Cloning and characterization of MST, a novel (putative) serine/threonine kinase with SH3 domain. Oncogene. 1995 Apr 6;10(7):1447-51. PMID: 7731697.
53. Dorow DS, Devereux L, Tu GF, Price G, Nicholl JK, Sutherland GR, Simpson RJ. Complete nucleotide sequence, expression, and chromosomal localisation of human mixed-lineage kinase 2. Eur J Biochem. 1995 Dec 1;234(2):492-500. doi: 10.1111/j.1432-1033.1995.492_b.x. PMID: 8536694.
54. Dorow DS, Devereux L, Dietzsch E, De Kretser T. Identification of a new family of human epithelial protein kinases containing two leucine/isoleucine-zipper domains. Eur J Biochem. 1993 Apr 15;213(2):701-10. doi: 10.1111/j.1432-1033.1993.tb17810.x. PMID: 8477742.
55. Durkin JT, Holskin BP, Kopec KK, Reed MS, Spais CM, Steffy BM, Gessner G, Angeles TS, Pohl J, Ator MA, Meyer SL. Phosphoregulation of mixed-lineage kinase 1 activity by multiple phosphorylation in the activation loop. Biochemistry. 2004 Dec 28;43(51):16348-55. doi: 10.1021/bi049866y. PMID: 15610029.
56. Derry JM, Ochs HD, Francke U. Isolation of a novel gene mutated in Wiskott-Aldrich syndrome. Cell. 1994 Dec 2;79(5):following 922. Erratum for: Cell. 1994 Aug 26;78(4):635-44. PMID: 8001129.
57. Symons M, Derry JM, Karlak B, Jiang S, Lemahieu V, Mccormick F, Francke U, Abo A. Wiskott-Aldrich syndrome protein, a novel effector for the GTPase CDC42Hs, is implicated in actin polymerization. Cell. 1996 Mar 8;84(5):723-34. doi: 10.1016/s0092-8674(00)81050-8. PMID: 8625410.
58. Abdul-Manan N, Aghazadeh B, Liu GA, Majumdar A, Ouerfelli O, Siminovitch KA, Rosen MK. Structure of Cdc42 in complex with the GTPase-binding domain of the 'Wiskott-Aldrich syndrome' protein. Nature. 1999 May 27;399(6734):379-83. doi: 10.1038/20726. PMID: 10360578.
59. Daniels RH, Bokoch GM. p21-activated protein kinase: a crucial component of morphological signaling? Trends Biochem Sci. 1999 Sep;24(9):350-5. doi: 10.1016/s0968-0004(99)01442-5. PMID: 10470034.
60. Abo A, Qu J, Cammarano MS, Dan C, Fritsch A, Baud V, Belisle B, Minden A. PAK4, a novel effector for Cdc42Hs, is implicated in the reorganization of the actin cytoskeleton and in the formation of filopodia. EMBO J. 1998 Nov 16;17(22):6527-40. doi: 10.1093/emboj/17.22.6527. PMID: 9822598; PMCID: PMC1171000.
61. Pandey A, Dan I, Kristiansen TZ, Watanabe NM, Voldby J, Kajikawa E, Khosravi-Far R, Blagoev B, Mann M. Cloning and characterization of PAK5, a novel member of mammalian p21-activated kinase-II subfamily that is predominantly expressed in brain. Oncogene. 2002 May 30;21(24):3939-48. doi: 10.1038/sj.onc.1205478. PMID: 12032833.
62. Wen YY, Wang XX, Pei DS, Zheng JN. p21-Activated kinase 5: a pleiotropic kinase. Bioorg Med Chem Lett. 2013 Dec 15;23(24):6636-9. doi: 10.1016/j.bmcl.2013.10.051. Epub 2013 Oct 31. PMID: 24215894.
63. Yokoyama N, Miller WT. Purification and enzyme activity of ACK1. Methods Enzymol. 2006;406:250-60. doi: 10.1016/S0076-6879(06)06018-6. PMID: 16472662.
64. Rousseau V, Goupille O, Morin N, Barnier JV. A new constitutively active brain PAK3 isoform displays modified specificities toward Rac and Cdc42 GTPases. J Biol Chem. 2003 Feb 7;278(6):3912-20. doi: 10.1074/jbc.M207251200. Epub 2002 Dec 2. PMID: 12464619.
65. Jaffer ZM, Chernoff J. p21-activated kinases: three more join the Pak. Int J Biochem Cell Biol. 2002 Jul;34(7):713-7. doi: 10.1016/s1357-2725(01)00158-3. PMID: 11950587.
66. Yao D, Li C, Rajoka MSR, He Z, Huang J, Wang J, Zhang J. P21-Activated Kinase 1: Emerging biological functions and potential therapeutic targets in Cancer. Theranostics. 2020 Aug 1;10(21):9741-9766. doi: 10.7150/thno.46913. PMID: 32863957; PMCID: PMC7449905.
67. Qiu RG, Abo A, Steven Martin G. A human homolog of the C. elegans polarity determinant Par-6 links Rac and Cdc42 to PKCzeta signaling and cell transformation. Curr Biol. 2000 Jun 15;10(12):697-707. doi: 10.1016/s0960-9822(00)00535-2. PMID: 10873802.
68. Fukuoka M, Miki H, Takenawa T. Identification of N-WASP homologs in human and rat brain. Gene. 1997 Sep 1;196(1-2):43-8. doi: 10.1016/s0378-1119(97)00184-4. PMID: 9322739.
69. Martin GA, Bollag G, McCormick F, Abo A. A novel serine kinase activated by rac1/CDC42Hs-dependent autophosphorylation is related to PAK65 and STE20. EMBO J. 1995 Sep 1;14(17):4385. Erratum for: EMBO J. 1995 May 1;14(9):1970-8. PMID: 7556080; PMCID: PMC394523.
70. Johansson A, Driessens M, Aspenström P. The mammalian homologue of the Caenorhabditis elegans polarity protein PAR-6 is a binding partner for the Rho GTPases Cdc42 and Rac1. J Cell Sci. 2000 Sep;113 ( Pt 18):3267-75. PMID: 10954424.
71. Brajenovic M, Joberty G, Küster B, Bouwmeester T, Drewes G. Comprehensive proteomic analysis of human Par protein complexes reveals an interconnected protein network. J Biol Chem. 2004 Mar 26;279(13):12804-11. doi: 10.1074/jbc.M312171200. Epub 2003 Dec 15. PMID: 14676191.
72. Dormoy V, Tormanen K, Sütterlin C. Par6γ is at the mother centriole and controls centrosomal protein composition through a Par6α-dependent pathway. J Cell Sci. 2013 Feb 1;126(Pt 3):860-70. doi: 10.1242/jcs.121186. Epub 2012 Dec 21. PMID: 23264737; PMCID: PMC3619814.
73. Marques E, Englund JI, Tervonen TA, Virkunen E, Laakso M, Myllynen M, Mäkelä A, Ahvenainen M, Lepikhova T, Monni O, Hautaniemi S, Klefström J. Par6G suppresses cell proliferation and is targeted by loss-of-function mutations in multiple cancers. Oncogene. 2016 Mar 17;35(11):1386-98. doi: 10.1038/onc.2015.196. Epub 2015 Jun 15. PMID: 26073086; PMCID: PMC4800288.
74. Durgan J, Kaji N, Jin D, Hall A. Par6B and atypical PKC regulate mitotic spindle orientation during epithelial morphogenesis. J Biol Chem. 2011 Apr 8;286(14):12461-74. doi: 10.1074/jbc.M110.174235. Epub 2011 Feb 7. PMID: 21300793; PMCID: PMC3069449.
75. J. Cell Sci.: doi:10.1242/jcs.217869: Supplementary information. Pichaud F, Walther RF, Nunes de Almeida F. Regulation of Cdc42 and its effectors in epithelial morphogenesis. J Cell Sci. 2019 May 21;132(10):jcs217869. doi: 10.1242/jcs.217869. PMID: 31113848.
